# Structural gaps in referral and treatment pathways for gambling-related harm: a systematic review of health system responses using the antecedents–decision–outcomes framework

**DOI:** 10.3389/fpubh.2026.1823843

**Published:** 2026-06-19

**Authors:** Nathan Lakew, Philip Lindner

**Affiliations:** 1 Department of Clinical Neuroscience, Centre for Psychiatry Research, Karolinska Institutet, Solna, Sweden; 2 Stockholm Health Care Services, Region Stockholm, Stockholm, Sweden

**Keywords:** ADO framework, gambling disorder, gambling-related harm, health system readiness, public mental health, referral pathways, treatment systems

## Abstract

**Introduction:**

Gambling-related harm is increasingly recognized as a public mental health concern, yet referral pathways into formal treatment and support services remain poorly understood. Weak or absent referral structures may delay engagement with care and limit coordination across health and support systems. This review aimed to map structural and system-level factors shaping referral and treatment pathways for gambling-related harm. Particular attention was given to mechanisms influencing help-seeking, healthcare system readiness, and the extent to which gambling environments facilitate transitions into formal care.

**Methods:**

A systematic review was conducted across multiple databases covering literature published between 2014 and 2024. Studies were included if they examined help-seeking, referral pathways, service engagement, or system-level responses to harmful gambling. From an initial pool of 8,178 studies, 5,230 were screened, and 39 met the inclusion criteria. Findings were analyzed using ADO-guided thematic synthesis as the primary analytical approach, complemented by abstract-based descriptive bibliometric mapping.

**Results:**

Ten core themes were identified across the ADO phases. Antecedent-level findings highlighted barriers related to service visibility, system readiness, and the framing of responsibility. Decision-phase themes included help-seeking triggers, digital interventions, and the role of referral gatekeepers. Outcome themes focused on treatment engagement, dropouts, and user experiences of treatment. Across the included peer-reviewed literature, few studies reported structured referral pathways between gambling platforms and healthcare services, which shows an important gap in the documented evidence base. Responsibility framing emerged as a cross-cutting influence shaping how institutions recognize and respond to gambling-related harm.

**Conclusion:**

The evidence base remains limited, with most studies descriptive in nature. The findings highlight structural weaknesses in current referral and treatment ecosystems, particularly the absence of formalized pathways linking gambling environments to healthcare systems. Addressing these gaps will require stronger coordination between gambling operators, healthcare services, and regulators, alongside implementation research to develop and evaluate system-level referral models.

## Introduction

Gambling-related harm is increasingly recognized as a multifaceted public health challenge that affects not only individuals who gamble but also families, communities, and healthcare systems (1). Epidemiological studies estimate that between 3.5–7% of the general population show signs of problem gambling, with 0.5–3.5% meeting diagnostic criteria for gambling disorder (2). Despite well-documented evidence supporting the effectiveness of treatment interventions, engagement with formal mental healthcare remains low. For example, Bijker et al. (3) reported that only one in 25 moderate-risk gamblers and one in five individuals with disordered gambling have sought help, while other estimates suggest that treatment uptake may be as low as 10% (4).

A range of barriers can hinder individuals experiencing gambling-related harm from seeking treatment (5). Over the past two decades, gambling has transformed into a highly accessible and diverse online activity, posing challenges for regulators and mental health services to effectively reach vulnerable populations (6). Patient-specific factors such as shame, denial of gambling problems, or reluctance to seek help are often described as internal barriers (7). Structural and systemic barriers may also impede access to care, including fragmented services, variations in referral protocols, and limited awareness of treatment options among both patients and primary care professionals (8, 9).

The treatment landscape is further shaped by the framing of responsibility—i.e., the attribution of accountability, the scope of institutional obligations, and the mechanisms for enforcement—which in turn dictate the design of the care delivery system (10). In this review, we define framing of responsibility as how gambling-related harm is explained across different domains of practice and discourse in, for example, public policy and regulation (political discourse), corporate and marketing communication (industry framing), and clinical or healthcare settings (treatment discourse). The dominant use of Responsible Gambling (RG) language, for instance, has been criticized for creating structural barriers and reinforcing stigmatizing messages that discourage help-seeking (11). Framing, therefore, can capture how responsibility for preventing or addressing harm is attributed to individuals, gambling operators, or wider systems and institutions.

In addition, there is growing concern about the absence of clear referral pathways between online gambling platforms and formal healthcare systems. A formal treatment represents a structured healthcare or therapeutic service provided by licensed or accredited professionals (e.g., public healthcare systems, specialized clinics/social care services, helpline-linked counseling, and professionally guided digital programs). This definition distinguishes formal treatment from self-help tools or industry-hosted RG features, which typically operate outside the healthcare system. Recent audits of online gambling platforms reveal that (12) although RG tools are increasingly implemented, operator-led efforts to channel users toward formal treatment services remain limited and largely unregulated. In contrast to the extensive interest in the clinical and psychological aspects of gambling disorder, the practical pathways through which those experiencing harm are referred to formal care remain poorly understood.

Against this background, we conducted a framework-based systematic review complemented by descriptive bibliometric mapping to examine structural gaps in referral and treatment pathways for gambling-related harm. The review examines the systems, mechanisms, and framing practices that shape how individuals are referred to—and supported within—formal treatment services. Particular attention is given to the channelization of players from online gambling environments into formal care, the readiness of healthcare systems to respond, and the processes that influence treatment engagement and outcomes. This review adheres to the PRISMA 2020 guidelines for systematic reviews (see Additional File 4) and address the following research questions:

- RQ1: What structures exist—or are missing—to enable referral of individuals experiencing gambling-related harm from operators’ platforms to formal treatment or support systems?
- RQ2: What organizational and structural features characterize treatment systems for individuals seeking support outside gambling operator environments?
- RQ3: How is responsibility for gambling-related harm framed across individuals, the gambling industry, and healthcare systems?

## Methods

### Review design

There exist different bases for conducting systematic reviews: domain-based, theory-based, method-based, and meta-analytical-based reviews (13). This study adopts a domain-based systematic review approach. Domain-based reviews provide a means to capture state-of-the-art research within a specific field while enabling the integration of multiple synthesis methods (14). Such reviews may adopt structured, framework-based, bibliometric, or hybrid designs that combine elements of multiple approaches (15).

The current study implements a hybrid systematic review design that integrates descriptive bibliometric mapping with a framework-based thematic synthesis. This dual-method approach provided complementary perspectives on the literature by combining broader conceptual mapping with in-depth thematic interpretation. Customarily described as a “well-done” review (16), the framework-based synthesis served as the primary analytical approach to examine how barriers, help-seeking decisions, and engagement outcomes are interconnected in shaping system-level responses to gambling-related harm. The bibliometric component was incorporated as a complementary descriptive tool to map the intellectual structure, keyword co-occurrence, and conceptual clusters of the broader field (*n* = 252 abstracts). This provided a descriptive overview of the field’s conceptual landscape, including thematic clustering and conceptual relationships across the literature. By integrating these two methods, the study used bibliometric mapping to contextualize and support the substantive interpretation of the thematic findings.

We adopt the Antecedents–Decision–Outcomes (ADO) framework developed by Paul and Benito (13), which provides an analytical lens to map out processes and mechanisms across a broader system continuum. In our context, Antecedents refer to the structural or individual conditions that shape awareness of harm and readiness to seek or offer support; Decisions encompass the triggers, barriers, or system responses involved in initiating treatment or referral; and Outcomes capture whether individuals are successfully linked to formal treatment, remain engaged, and experience meaningful support. The review uses the ADO framework to identify key constructs across each phase and analyze how responsibility framing shapes access to treatment pathways. As such, framing is treated both as a topic of interest (RQ3) and as a cross-cutting interpretive lens across all ADO phases.

### Search strategy and data sources

**Figure 1:**
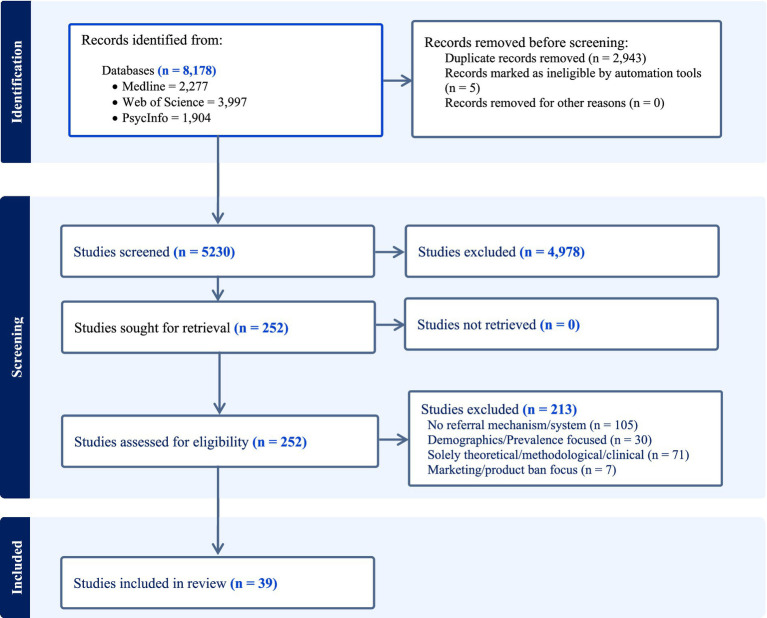
PRISMA framework for identification of studies via databases. Bold text in the figure represent results at that particular stage. E.g., n = 252 is the result of number of studies at that stage.

The search strategy was developed in collaboration with librarians at the Karolinska Institutet University Library, with the primary strategy constructed in Medline (Ovid). The review followed the Preferred Reporting Items for Systematic Reviews and Meta-Analyses (PRISMA) guidelines for article identification and screening (see Figure 1). Only peer-reviewed journal articles were included in the review. Literature searches were conducted across three databases—Medline, Web of Science, and PsycInfo—covering the period from January 2014 to May 2024. The search strategies were adapted to each database with the support of the Polyglot Search Translator (17). Duplicate records were removed following the method described by Bramer et al. (18), with an additional step to compare DOIs for residual duplication. All strategies were peer-reviewed by an independent librarian prior to execution.

The search strategy targeted two core conceptual blocks—healthcare systems and gambling operators—with keywords combining variations of gambling-related terms and concepts such as primary care, mental health services, public health systems, policy framing, customer protection, operator responsibility, harm reduction, industry strategy, and Corporate Social Responsibility (CSR). For each concept, a combination of Medical Subject Headings (MeSH) and free-text terms was used across databases. The full search strategies for each database are provided in Additional File 1.

### Eligibility criteria and screening process

After initial deduplication and eligibility filtering, all studies were imported into the Covidence© collaborative review platform for screening and distributed among the reviewers. After conducting title and abstract-based screening, 252 articles were chosen for a full-text review. Studies were included if they examined gambling-related harm concerning referral, treatment seeking, or engagement with formal support systems (i.e., healthcare services, helplines, or structured therapy). In addition, studies that address organizational structures, system readiness, or mechanisms that link individuals from gambling environments to formal care were included. Interventions initiated by gambling operators were included only if they involved pathways toward formal treatment or external referral. Studies using any methodological approach, randomized, observational, qualitative, or conceptual, were eligible if they examined mechanisms related to referral, linkage, or engagement with treatment and support systems. Trials involving participants not actively seeking help were also retained if they provided conceptual insight into early-stage structural barriers that precede formal referral or treatment entry.

Studies were excluded if they (1) focused solely on evaluating clinical interventions (e.g., CBT, motivational interviewing, or pharmacological intervention) without addressing referral or system structures; (2) discussed theoretical frameworks, philosophical discussions, or methodological issues without applied system-level analysis; (3) evaluated population-level interventions without treatment linkage (e.g., Socio-demographic risk profiling or general prevention campaigns); or (4) focused on gambling-related marketing, advertising restrictions, or platform design without connection to formal care or referral pathways. Screening and extraction were conducted using the Covidence platform with predefined inclusion and exclusion criteria. Title and abstract screening were performed by one reviewer, with uncertain cases discussed collaboratively among the review team. Full-text inclusion was verified by two reviewers. Data extraction and ADO-based thematic coding were conducted using predefined extraction criteria and iterative coding discussions to support consistency in interpretation across studies.

### Data extraction and synthesis

Data extraction from the 39 included studies was carried out using a structured ADO-aligned template by the first author to support consistency in interpreting framework dimensions and thematic patterns. Extraction fields captured bibliographic details, methodological description, study context, ADO phase tagging, referral type, framing type, and key findings (see Supplementary Table 2 in Additional File 2). The structured extraction sheet guided the deductive framework-based synthesis and thematic analysis, while iterative discussions among the review team were used to refine interpretation across ADO phases. NVivo 15 and Excel were used to organize and code the extracted data.

### Rapid quality appraisal

To assess evidential strength, an independent rapid quality appraisal was conducted across all 39 studies (see Additional File 3). Given the heterogeneity of the included literature, a customized rapid appraisal approach was used instead of a single standardized quality assessment tool. The appraisal focused on evaluating the robustness and transparency of research addressing referral and treatment system structures for gambling-related harm. Three topic-specific criteria and one additional measure of data transparency were applied:

1) Clarity of referral or pathway definition
2) Adequacy of outcome measurement
3) Reporting of attrition or follow-up
4) Transparency of data source and sampling (used as a proxy for study trustworthiness)

Each criterion was assessed using High, Low, or N/A labels to contextualize evidential strengths and reporting quality across the literature rather than to determine study exclusion or quantitative weighting. Across the evidence, studies demonstrated high data transparency and clear reporting of data sources, sampling methods, and analytic approaches. Strong performance was also observed for outcome measurement, particularly in studies evaluating treatment engagement or behavioral change. However, greater variation was visible in the definition of referral mechanisms and follow-up reporting, reflecting conceptual gaps.

### Descriptive bibliometric analysis

Next, a bibliometric analysis was performed using both keyword co-occurrence mapping and abstract text co-word clustering. Keyword co-occurrence was explored using VOSviewer to descriptively examine frequently linked terms and conceptual proximities across the included studies. In parallel, a Python-based co-word analysis was applied to abstract texts using Term Frequency–Inverse Document Frequency (TF-IDF) weighting and cosine similarity to explore semantic relationships between terms. TF-IDF is a common weighting method used in text mining to identify terms that are contextually distinctive within a document corpus (19, 20). Visualization was performed using matplotlib, Seaborn, and VOSviewer to generate descriptive network graphs and heatmaps. The bibliometric component included both broader keyword co-occurrence mapping across the 252 abstracts considered during full-text screening and a focused descriptive exploratory mapping of the 39 included studies to support contextualization of the ADO framework synthesis.

### ADO framework-based thematic synthesis

For the primary analysis, each of the 39 included studies was coded using a structured data extraction template aligned with the ADO phases systematically to identify conditions that influence harm recognition (Antecedents), referral triggers (Decision), and treatment linkage or engagement success (Outcomes). Full-text coding was conducted using NVivo 15, where codes were organized hierarchically under each ADO phase and refined deductively as themes emerged. The coding process involved iterative refinement of thematic categories while consistently adhering to the three ADO phase categories to support analytical consistency and coherence. This analysis served as the conceptual backbone of the review and was triangulated with bibliometric mapping to report key constructs, gaps, and cross-cutting patterns (e.g., framings). See Supplementary Figure 1 in Additional File 2 for a general overview of the review process and analysis method.

## Results

### Study selection and characteristics

A total of 8,178 records were identified (Medline: 2,277; Web of Science: 3,997; PsycInfo: 1,904). After de-duplication, 5,230 unique records were screened at the abstract level. Of these, 252 full-text articles were reviewed, and 39 met the inclusion criteria (see Figure 1 for PRISMA flow). Published between 2014 and 2024, study contexts ranged from public healthcare and helplines to digital platforms, covering both traditional and emerging intervention modes. Detailed study characteristics, including design, country, and ADO alignment, are presented in Supplementary Tables 1, 2 in Additional File 2.

### Bibliometric analysis result

#### Keyword co-occurrence mapping

**Figure 2:**
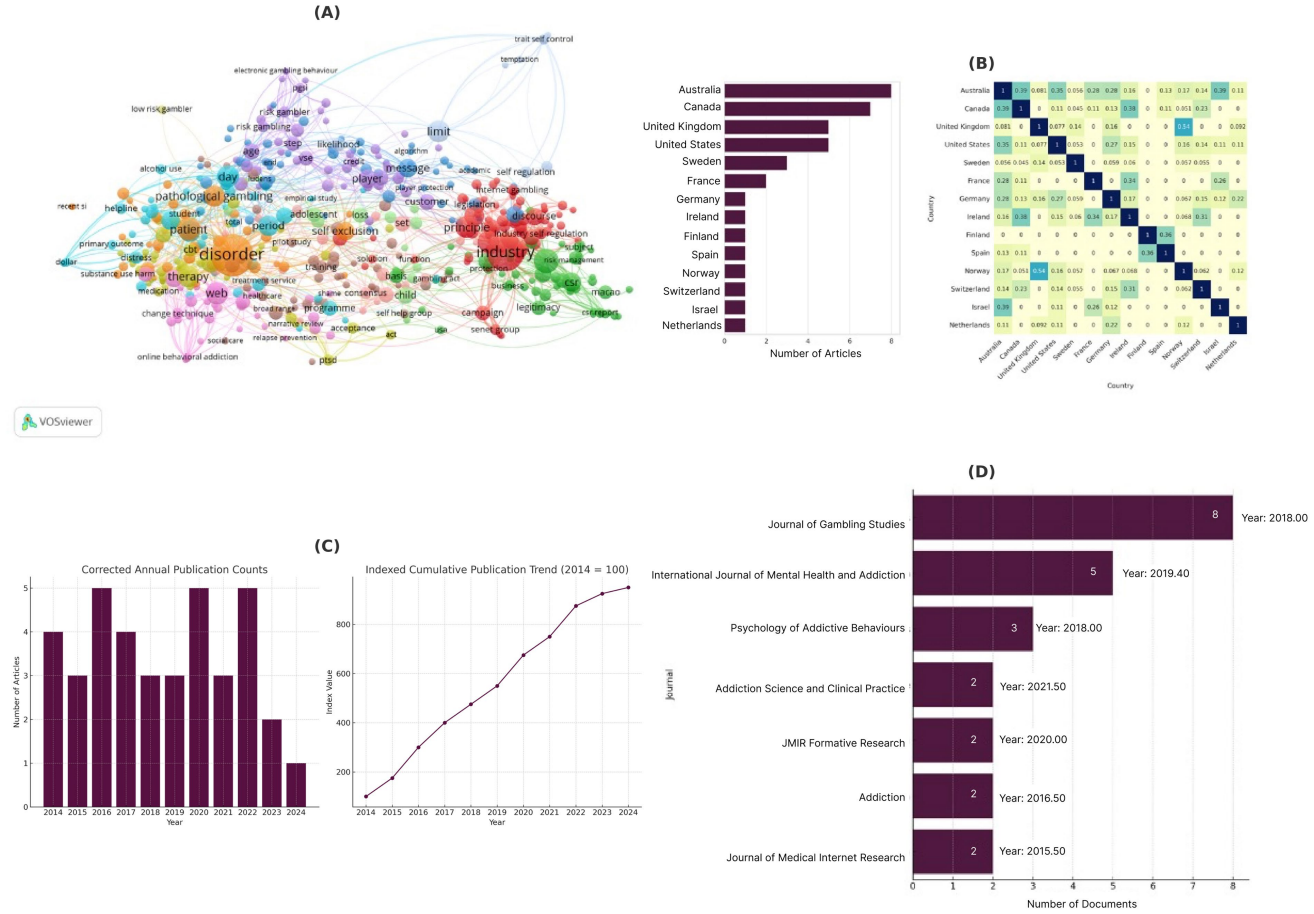
**(A)**. Keyword co-occurrence of 252 abstracts showing thematic clusters in the literature; **(B)** Research output by country (left) and thematic similarity based on conceptual focus (right); **(C)** Annual and indexed cumulative publication trend; **(D)** Source clustering and centrality.

For a broader view, a keyword co-occurrence analysis was conducted on a larger dataset of 252 abstracts considered for full-text review. The resulting map revealed six major clusters (see Figure 2A). The orange cluster focused on clinical and diagnostic discourse with terms such as therapy and CBT. A large red cluster centered on industry-led framing featuring keywords such as CSR, discourse, legitimacy, and campaign. The green cluster reflected policy and regulatory themes, particularly related to national governance, while the blue cluster emphasized digital tools and behavioral messaging (e.g., limit, player tracking, message). Additional smaller clusters addressed risk assessment, self-regulation, and consumer monitoring strategies. This structural distribution shows dominant field-level concerns across the 252 abstracts; though the map serves as an exploratory tool, it highlights the relatively limited literature focused on evaluated referral structures or direct system linkage.

#### Geographical clustering and conceptual focus

Narrowing our focus to the 39 included studies, we conducted a country-level bibliometric analysis on publication volume and themes (see Figure 2B). A geographical analysis showed a concentration of research activity in Anglophone and high-income countries: Australia, Canada, the United Kingdom, and the United States (see Figure 2B/1). These countries accounted for over two-thirds of the reviewed literature. To explore conceptual patterns, a similarity matrix was constructed using TF-IDF vectorization of each country’s research focus and dominant journal outlets, followed by cosine similarity (see Figure 2B/2). The resulting heatmap reveals that Australian and Canadian studies often focused on helpline models, digital self-exclusion tools, and public health framing; UK and Nordic articles emphasized policy-level discussions and system integration challenges. Notably, contributions from lower-income and non-Western countries were largely absent from the literature, a pattern previously noted in gambling harm research (21).

#### Publication trends

The indexed cumulative trend of publications between 2014 and 2024 shows overall growth in research related to gambling treatment and referral systems (see Figure 2C/2), yet this growth has not followed a steady or linear trajectory. Instead, research on gambling-related treatment referral and system-level responses appears fragmented, with inconsistent attention over time (see Figure 2C/1). This ‘irregular’ trajectory may suggest that the field is still in formation, at times with thematic depth, but lacking a cohesive research program or long-term investment in the topic. This is despite the clear shift of gambling to online platforms (22), which has also created significant opportunities to develop structured referral pathways into formal treatment. Moreover, since such structured channelization mandates greater participation from operators, progress in this area needs system-level regulatory pressure as well as standardized referral protocols that may have faced commercial resistance (23).

#### Source clustering and centrality

Using Python (Matplotlib), a source-level bibliographic coupling map was generated to assess citation similarity across journals (see Figure 2D). Based on shared references between articles, the analysis suggested that the Journal of Gambling Studies emerged as the most central source in the dataset. The overall coupling strength across sources, however, was low, which may reflect a fragmented intellectual landscape in the area. Among the other top contributing journals, the International Journal of Mental Health and Addiction accounted for five articles, while Addiction Science & Clinical Practice, the Journal of Medical Internet Research, and Psychology of Addictive Behaviors each contributed two. These journals also exhibited minimal bibliographic connectivity, suggesting limited theoretical and empirical integration across the literature addressing referral mechanisms and system-level treatment responses.

#### Thematic mapping (abstract-based co-word analysis)

The co-word network map generated using VOSviewer revealed naturally occurring clusters of semantically related terms (Supplementary Figure 2 in Additional File 2). These clusters illustrated broader thematic zones such as referral pathways and system structures, harm reduction and public health framing, intervention delivery formats, and treatment outcomes. In addition, the TF-IDF weighted analysis highlighted terms such as gambling, harm, treatment, and support as highly salient across the abstracts, reflecting their centrality in the literature (Supplementary Figure 3 in Additional File 2). In contrast, terms like platform and operator appeared only as peripheral.

### ADO framework-based thematic synthesis result

Building on the conceptual landscape, the main synthesis used the ADO framework to organize a more granular thematic analysis of the full-text articles. This was carried out in two steps: (1) structured data extraction using manifest content analysis of all 39 studies (see Supplementary Table 3 in Additional File 2), and (2) in-depth thematic coding in NVivo to capture latent themes across the ADO phases. The resulting thematic constructs are presented in Table 1. To illustrate the hierarchical structure of these themes, a project map was generated for each ADO phase using NVivo’s code tree (see Supplementary Figure 3 in Additional File 2).

**Table 1:** Thematic constructs categorized by ADO framework (based on full-text coding of 39 studies).

ADO framework	Theme	Description
Antecedents	Recognition and awareness of harm	Focuses on how harm is identified and understood by individuals, families, and service providers, including the influence of stigma, normalization, and denial.
	Framing of responsibility	Explores how responsibility for gambling harm is attributed across individuals, systems, and operators, and in effect, shapes intervention responses.
	Visibility and accessibility of services	Examines how service awareness, accessibility, and structural fragmentation affect help-seeking.
	System and Operator Readiness	Assesses institutional and operator-level capacity, training, and infrastructure to enable support and referral.
Decision	Crisis as a trigger	Analyzes how acute financial or emotional crises prompt help-seeking in the absence of preventive mechanisms.
	Digital platform-based interventions and prompts	Looks at how digital tools, nudges, and interfaces influence decisions to seek formal healthcare support.
	Referral pathways and gatekeepers	Describes the role of helplines, clinicians, and informal actors in guiding individuals toward care.
Outcomes	Engagement and retention in treatment	Examine factors that support sustained involvement in care, including user-service alignment and continuity.
	Drop-off and referral failure	Highlight points of disengagement or breakdown in the referral process after initial contact.
	Reported outcomes and perceived impact	Reviews how effectiveness, satisfaction, and recovery are measured and experienced across settings.

#### Antecedents of the ADO framework

The antecedent phase outlines the conditions that enable recognition and response to harmful gambling. This includes how harm is framed, how visible and accessible support systems are, and the degree to which individuals, operator platforms, and institutions are prepared to facilitate help-seeking. Four themes were identified in the antecedent phase.

##### Theme one: Recognition and awareness of harm

A critical antecedent to help-seeking is the recognition of gambling-related harm by both the individual and the system. Yet we found that recognition is often delayed or obscured by a combination of personal, social, and institutional factors. Psychological barriers such as denial, stigma, and shame repeatedly emerge as key inhibitors of help-seeking (24), while the absence of systematic screening and professional awareness limits opportunities for early detection (25). In addition, the review shows that individuals with lived experience of psychological distress are more likely to perceive gambling as treatable (26), and unsolicited inclusion in an intervention may itself prompt self-recognition of harm. However, readiness to engage remains low among those not actively seeking support (27). This suggests that recognition often precedes but does not necessarily lead to help-seeking behavior.

At the institutional level, recognition gaps persist due to the low visibility of problem gambling in healthcare, where even informed clinicians rarely conduct routine screening (28). This reflects uncertainty about responsibility and weak institutional mandates (28, 29). Recognition also appears context-dependent (e.g., gambling modality, perceived severity, and how harm is framed within policy and clinical discourse) (29, 30). While both individuals and systems may recognize the existence of gambling-related harm, the absence of structured pathways into formal healthcare often prevents this awareness from translating into early intervention.

##### Theme two: Framing of responsibility

The review finds that responsibility attribution for gambling-related harm (i.e., individual, corporate, or systemic) shapes both help-seeking behavior and intervention design. Reno model-oriented studies have been criticized for prioritizing personal responsibility while downplaying structural accountability, thereby minimizing the roles of operators and, at times, regulators, in harm prevention (31, 32). When responsibility is framed narrowly around individual willpower or pathology, interventions tend to focus on self-exclusion, personal motivation, or compliance (e.g., with therapy). In addition, at the individual level, internalized blame can reinforce stigma and reduce help-seeking, particularly when treatment is perceived as an admission of personal failure (24, 29). In contrast, when harm is framed as a structural or socially distributed issue, emphasis shifts toward proactive screening, operator accountability, game design issues, and cross-sector coordination (23, 32). Theme two studies illustrate that framing influences both the discourse on gambling harm and the organization of treatment and prevention systems.

##### Theme three: Visibility and accessibility of services

We also found that the visibility of treatment options plays a critical role in shaping early engagement. Key barriers in this context include limited professional awareness, service fragmentation, and unclear referral pathways. This is particularly acute for the youth population, where problem gambling is often deprioritized (33). Clinicians frequently report poor visibility of referral routes and limited knowledge of available services (34), while treatment systems are described as under-resourced and difficult to navigate (35).

Evidence from evaluations of internet-based interventions suggests that accessibility, therapist support, and system visibility play a key role in shaping users’ willingness to engage (36). In addition, a lack of health providers’ integration with the gambling support network, bureaucratic complexity, and regional disparities in implementation methods can leave individuals to navigate parallel or disconnected systems (24, 37). Theme three findings suggest that limited-service visibility is not merely a logistical challenge but a systemic bottleneck that delays timely help-seeking and disrupts treatment continuity.

##### Theme four: System and operator readiness

Readiness encompasses institutional capacity, staff training, organizational protocols, and operators’ willingness to engage harm reduction. The review articles in this theme show significant gaps in the field, including the absence of tools and formal directives among clinicians (34), underused screening tools (38), structural issues such as lack of integration (39), and inconsistent public health mandates (40). Lopez-Gonzalez et al. (41) also noted that current gambling treatments are inadequate in addressing the needs of online gamblers, particularly young individuals, due to their age, tech savviness, resistance to abstinence, and the unique structures of skill-based online gambling.

Readiness is also shaped by cultural fit (26, 42), staff specialization (9), helpline efficacy (43), and expert-defined competencies (44). While many of these gaps reflect systemic inertia, others reveal deeper policy alignment issues. As Regan et al. (23) emphasize, readiness is less a matter of technical capacity and more a political and ethical orientation toward public health responsibility. Regan et al. (23) argue that readiness is less a matter of technical capacity than of political and ethical orientation toward public health responsibility. Their approach emphasized the need for cultural tailoring, stakeholder co-design, and cross-sector collaboration. Theme four findings position readiness as a multidimensional construct encompassing contextual alignment, political will, and clinical competence.

#### Decisions of the ADO framework

The decision phase focuses on the moment of action when individuals move from recognition to response. It captures the triggers, mechanisms, and referral processes that prompt help-seeking, whether initiated by the individual, gambling platforms, or institutional actors. We found three themes within the decision phase.

##### Theme five: Crisis as a trigger

Help-seeking for gambling-related harm is most often precipitated by acute crisis rather than gradual recognition or preventive awareness. Across studies, individuals typically seek help after reaching a point of severe emotional, financial, or relational breakdown. Common triggers include debt accumulation, eviction risk, partner conflict, and psychological distress such as guilt or suicidal ideation (24, 37, 43). Since these crises frequently coincide with shame and loss of control, help-seeking emerges less as a voluntary act of insight and more as a compelled response to impending collapse.

However, not all crises translate into successful treatment. Evidence shows (45) that crises grounded in tangible loss (e.g., financial or family disruption) are more likely to result in treatment follow-through than diffuse emotional turmoil. The effectiveness of crisis response depends on system-level factors such as the availability, training, and empathy of frontline staff, which also influence whether individuals move from acute distress to structured care. Crisis as a trigger theme reveals systemic unpreparedness and the predominance of reactive over preventive referral models.

##### Theme six: Digital platform-based interventions and prompts

Digital interfaces can play a role in triggering help-seeking or interrupting harmful gambling behavior. These include operator-based pop-up messages, self-exclusion systems, online self-help programs, helpline-based chats, and structured digital counseling platforms. Yet implementation and impact vary considerably. Some studies (12) report that operator-provided harm-reduction tools are inconsistently applied across platforms, while others (30) note that users often perceive them as insincere when not connected to transparent referral mechanisms. Studies on digital health programs report mixed outcomes, with engagement influenced less by the intervention itself than by its perceived fit with users’ needs and contexts. Tools that feel intuitive and supporting autonomy foster use, while effortful platforms drive dropouts (46, 47). In addition, when interventions targeted non help-seeking participants, high attrition appeared linked less to program design than to users’ limited motivation or readiness for formal care (27).

##### Theme seven: Referral pathways and gatekeepers

Referral to gambling treatment often relies on gatekeepers such as General Practitioners (GPs), helpline counselors, operators, support groups, and mental health professionals. These actors can play a critical intermediary role in accessing formal treatment, yet our review suggested that referral processes tend to be informal and poorly defined. Evidence from a Swedish primary care study (48) shows that brief interventions can be effective when delivered by trusted clinicians within existing appointments, but unclear screening procedures and low follow-up rates often undermine continuity. Similarly, Nehlin et al. (49) highlight growing interest in screening and referral models within healthcare but also point to structural barriers such as inadequate training, lack of standardized protocols, and poor post-contact coordination. Pilot work by Reid et al. (50) illustrate how community-based screening by GPs and social service providers can activate referral pathways when co-designed to ensure cultural responsiveness.

Across the studies, the relational dimension of referral system repeatedly emerges as central to success. For example, mutual aid pathways such as Gamblers Anonymous (GA) can form a self-initiated pathway into support. Similarly, Valdivia-Salas et al. (45) reported that the quality of interpersonal interaction, including trust, empathy, and motivational reinforcement, can strongly influence whether individuals act on a referral. Structured helpline interventions that integrate relational continuity demonstrate higher follow-through rates than purely informational referrals (51).

Notably, operator-initiated referral systems linking players to formal treatment were rarely documented within the included literature. While gambling platforms increasingly provide RG tools and help links, formal referral pathways into healthcare or support systems remain largely underdeveloped. The resulting landscape reflects partial connectivity, where referrals can be initiated but remain neither systematically implemented nor institutionally supported: a critical gap in linking individuals experiencing gambling harm to formal healthcare.

#### Outcome of the ADO framework

The outcomes phase examines what follows initial contact: whether individuals are successfully linked to formal treatment services, remain engaged, and receive meaningful support. It also reflects the consequences of earlier stages, including successful trajectories, points of drop-off, and disengagement. Three themes were identified in the outcome phase.

##### Theme eight: Engagement and retention in treatment

Across the literature, structured and personalized approaches consistently outperform generic or one-size-fits-all formats. For instance, digital programs integrating therapist interaction significantly improve adherence and gambling reduction compared to unguided formats (47), while motivational techniques integrating in-person counselling strengthen commitment among clients (9, 52). Equally, the adaptability of programs to users’ lived experiences influences long-term retention. Integrated and context-sensitive models (e.g., shelter-based programs or culturally tailored screening in primary care settings) (50) demonstrated that peer support can translate into better engagement with intervention as well as measurable outcomes (e.g., improved financial control) (53). Emerging strategies such as Contingency Management models also show promise, particularly when co-designed with participants, though ethical and financial feasibility remain a concern (54). Overall, programs that align therapeutic structure with user autonomy, trust, and contextual relevance are better equipped to sustain participation, which suggests that engagement improves when services are flexible and structurally embedded in users’ contexts.

##### Theme nine: Drop-off and referral failure

Despite recognition of harm or initial contact with services, many individuals fail to remain in treatment as a result of both system and individual-level factors. In one national digital treatment program, dropout was highest among younger users, individuals with weak social networks, and those with low readiness to change (46). Crucially, longer waiting time was linked to higher dropout rates, which underscores how service design factors can significantly undermine retention. Luquiens et al. (27), for example, reported a 97% dropout rate from a digital CBT program targeting non-help-seeking gamblers, particularly among those in guided therapy. The authors interpreted this attrition as evidence that engagement readiness must precede structured treatment formats and that perceived autonomy and anonymity may be prerequisites for participation among disengaged users.

Similar patterns were observed in helpline-based and web-linked referrals. Valdivia-Salas et al. (45) found that fewer than half of helpline clients attended their first counseling session, while Darbeda et al. (43) noted that web-based gamblers were less likely to receive referrals despite comparable severity levels, suggesting under-identification of digital risk profiles. These studies point to a structural paradox: while care based on remote modalities can increasing accessibility through digital means, they often lack the relational continuity and motivational scaffolding necessary for sustained participation.

##### Theme ten: Reported outcomes and perceived impact

Evaluations of treatment outcome studies indicate that structured interventions involving therapist support consistently yield the greatest reductions in gambling severity. A review of 21 RCTs shows that cognitive-behavioral programs are most effective with ongoing therapist involvement, while brief or self-help formats suit milder cases but struggle to maintain long-term engagement (48, 55). Outcome variation also appears to be influenced by demographic and contextual factors. Age of onset, for instance, has been associated with variation in treatment experience and recovery trajectory, suggesting that interventions may benefit from tailoring to life stage and gambling modality (56).

Finally, treatment outcomes are strongly influenced by the degree to which interventions align with user needs. When tailored appropriately, even single-session formats can produce outcomes comparable to multi-session CBT (57). In more complex cases involving co-occurring mental health issues, integrated models that involve healthcare and social services have demonstrated clinical effectiveness and practical feasibility (58). The findings from theme ten highlight that treatment success is contingent on both therapeutic technique and on alignment between users’ needs, intervention structure, and broader system integration.

## Discussion

### Principal findings

This systematic review provides a systems-level perspective on how referral and treatment pathways for gambling-related harm are structured across digital gambling environments and healthcare services. Three overarching findings emerged. First, across the 39 included studies, there was limited evidence of structured referral pathways connecting online gambling environments with formal care services (RQ1). Although many platforms deploy RG tools, few studies have reported formalized mechanisms that transition individuals from these tools into professional treatment. Second, once users initiate help-seeking, they encounter healthcare systems characterized by fragmentation, such as inconsistent screening practices and limited integration of gambling harm into broader mental health and addiction services (RQ2). Third, a dominant framing of gambling harm as a personal responsibility persists across institutional and policy domains (RQ3). While a small number of studies introduced alternative framings grounded in public health or structural accountability, these approaches remain underdeveloped in terms of practical implementation.

### Antecedents: missed opportunities for early intervention

Across the literature, we found that formal treatment remained difficult to access unless individuals already self-identified as needing help. The absence of screening tools, workforce protocols, and standardized care pathways contributed to missed opportunities for harm detection during earlier stages of problem development. These patterns were strongly shaped by framing, with a recurring emphasis on individual responsibility that prioritized personal readiness over system design. Overall, the antecedent findings point to a misalignment between the nature of gambling-related harm, often characterized by avoidance, ambivalence, and shame, and a support system that relies on individuals to initiate contact. This disconnect contributes to delayed recognition and missed opportunities for early intervention, especially in settings where routine care or platform-based screening could serve as an effective prevention tool.

### Decision points: reactive systems and limited gateways

Decision-making around help-seeking indicates that current referral models are primarily reactive, often trigged by acute financial or emotional collapse. The absence of formalized referral mechanisms from gambling platforms meant that users encountering digital RG tools were largely left to navigate formal support options independently. A few studies highlighted more collaborative or system-led approaches, such as helpline triage protocols or clinician-guided screening, which shifted the burden of decision-making away from the individual and toward shared responsibility. However, these models remained peripheral and were rarely integrated with digital gambling environments. Framing remained a critical factor in shaping these moments of action, or inaction. Many interventions positioned the gambler as solely responsible for initiating treatment, with support tools offered as optional features rather than embedded within a broader care trajectory. As such, the current system relies on personal crisis as the primary trigger for help-seeking, which leaves many opportunities for earlier intervention unrealized.

### Outcomes: disengagement and structural drop-off

The outcome phase revealed a consistent lack of systemic support for sustained treatment engagement. The absence of formal handover mechanisms between care providers created weak continuity and placed the burden of navigating care on the individual. Personalized and relationship-based approaches were found to support longer engagement, yet such practices were rarely standardized or mandated. Framing again shaped how outcomes were conceptualized and evaluated. Many studies defined success primarily in behavioral terms (e.g., gambling activity or abstinence). This narrow framing makes institutional accountability ambiguous while subtly reinforcing the perception that treatment failure is a result of personal shortcomings.

### Identified issues and gaps

This review identifies persistent gaps that constrain system-level responses to gambling-related harm. First, there is very limited peer-reviewed research evaluating channelization pathways from gambling operators into formal healthcare systems. Despite widespread implementation of RG tools across operators, little is known about their effectiveness in triggering referral or treatment engagement. Second, few studies offer practical guidance on how healthcare systems can build referral protocols or coordinate across departments. Third, the structural design of gambling healthcare systems remains underexplored. The organizational readiness of healthcare systems (e.g., staffing, mandate, and service integration) is rarely examined in detail, leaving unclear how services could absorb referrals even if they were initiated. Beyond general mentions of fragmentation or poor screening, few studies examine how mandates, departmental roles, or integration with addiction services shape access for individuals with gambling-related harm.

Finally, while critiques of the Reno model are abundant, few studies take the next step of exploring how a public health orientation could be operationalized through regulatory mandates, formal referral protocols, or healthcare system readiness in terms of staffing and integration of different services. In contrast, the gambling field in general has made significant progress in developing complex detection models for identifying risky gambling behavior and tracking individual trajectories (e.g., machine learning approaches). However, without corresponding efforts to build system-level referral and support structures, such innovative approaches risk reinforcing the notion that harm arises ‘solely’ from individual behavior, while sidelining the need for product accountability and system-wide responses.

### Public mental health and system implications

The findings of this review point to several actionable areas where system-level reforms are needed to align gambling harm reduction with public health principles. A key implication of this review is the need to formalize referral pathways linking gambling environments to healthcare or support services. At present, structured mechanisms that enable direct transitions from gambling platforms to treatment systems are largely absent. As a result, individuals experiencing gambling-related harm often enter care only after acute personal or financial crises, including suicidal ideation, severe debt, or relational collapse. Developing formal referral infrastructures could help bridge this gap by enabling earlier engagement with support services. Such mechanisms may involve structured and legally mandated protocols that allow coordinated handoffs between gambling platforms and healthcare providers, supported by clinical triage and follow-up systems to ensure continuity of care.

In addition, the review highlights the need to strengthen health system readiness within mental health services. Evidence suggests that individuals experiencing gambling-related harm frequently present to mental health services with psychiatric comorbidities, creating opportunities for screening and referral to specialized care. However, fragmented service structures and limited system readiness often prevent these opportunities from being effectively utilized. Establishing earlier “soft-entry” points into care—e.g., through screening within primary care or community-based services—could support earlier detection, improve intervention outcomes, and reduce public healthcare costs. Accordingly, our findings highlight the need to embed gambling-related screening within routine mental health and addiction assessments, alongside improved training for frontline professionals, to better integrate responses to gambling-related harm into existing care pathways.

Third, there is a need for a coordinated national strategy to reduce fragmentation across service regions and ensure alignment between gambling regulation, public health planning, and treatment pathways. Clear national frameworks can support integrated responses across sectors and administrative levels to improve consistency and equity in care access. Fourth, regulatory frameworks must extend beyond voluntary compliance. Stronger mandates are needed to require outcome reporting, monitor operator practices, and enforce consequences for non-compliance. Regulators should also obligate gambling platforms to support independent research and maintain transparent systems for evaluating the effectiveness of their harm-reduction strategies. Finally, accountability must be improved through data. Systematic tracking of referral completion and treatment outcomes is currently lacking. A public health infrastructure should support routine data sharing between healthcare providers, operators, regulators, and researchers to monitor performance and inform continuous system improvement.

### Limitations

This review has several limitations. The database coverage was restricted to peer-reviewed journal articles indexed in Medline, PsycInfo, and Web of Science, which provided a broad multidisciplinary reach. However, this restricted focus may have excluded applied, policy-oriented, regulatory, or operator-led practices that have not yet been formally examined in academic research. In addition, the dual focus of the review—covering both healthcare-based referral mechanisms and potential referral pathways from operators to formal treatment—created an inherent trade-off in search scope and keyword precision. The inclusion of both domains required a broad conceptual range of search terms, while the specificity of the topic (formal referral structures) at times narrowed retrieval. As a result, certain adjacent studies on treatment-seeking, service evaluation, or system performance may not have been captured if they did not explicitly describe referral or linkage structures. Furthermore, the concept of formal treatment varies across jurisdictions and research contexts, with no clear consensus on when care formally begins (e.g., at screening, referral, or clinical intake). This ambiguity poses a broader challenge for reviews of this kind, where boundaries between prevention, support, and treatment consistently overlap in the literature.

Service-evaluation papers were excluded if referral or pathway mechanisms were not empirically examined, potentially omitting certain practice-based insights. In addition, the framework-based thematic synthesis involved interpretive coding decisions across ADO phases. Although structured extraction procedures, predefined coding categories, and iterative refinement processes were used to support consistency, some degree of interpretive subjectivity remains inherent in reviews of this kind. It should also be noted that this review focused exclusively on individuals experiencing gambling-related harm and did not include significant or affected others (e.g., family members, partners), whose own help-seeking trajectories and support needs are also critical. This exclusion represents an important limitation, as this group often acts not only as initiators of referral, but also as individuals who seek help for secondary harms, emotional distress, and financial or relational consequences associated with gambling. Finally, the bibliometric component was designed to serve as a complementary descriptive tool rather than a standalone bibliometric analysis. Given the focused sample of included studies, these outputs should be interpreted as an exploratory contextualization of conceptual patterns and fragmentation within the literature, not as exhaustive statistical representations.

### Directions for future research

Future research should prioritize evaluating how healthcare systems are organizationally structured to identify and respond to gambling-related harm. This includes testing different models that clarify departmental roles, improve triage, and integrate gambling harm screening into routine workflows. Additional research is also needed to explore patient needs across multiple levels to inform system-wide readiness strategies. For example, empirical research could assess the feasibility, effectiveness, and retention outcomes of real-time referral mechanisms linking gambling platforms to treatment services. Longitudinal studies that follow individuals through the referral and treatment pathway can help clarify which handoff protocols and coordination strategies actually facilitate engagement and recovery.

In parallel, scholarship should extend beyond critiques of individual responsibility framing to examine how public health framings can be operationalized in system design, policy mandates, and clinical practice. Methodologically, the field would benefit from implementation trials, post-implementation evaluations, and comparative studies across jurisdictions. From a policy and regulatory perspective, it is essential to establish robust evaluation criteria for duty-of-care initiatives, coupled with greater transparency in platform data and compliance reporting, to enhance accountability and inform future policy. Beyond identifying risky gambling behavior, duty of care regulation should also mandate that operators take active responsibility for facilitating referrals to formal treatment.

In this review, we focused exclusively on peer-reviewed literature. However, topics related to policy implementation, regulatory practice, and operator responsibility may also be reflected outside the academic literature. Future research should therefore examine grey literature, regulatory documents, and operator-facing materials to determine whether referral initiatives exist in practice but remain undocumented within peer-reviewed research. Finally, future research should also attend to significant others (e.g., family members or partners) who often experience secondary harm and may independently seek support. Their help-seeking behavior and referrals in the healthcare system warrant a dedicated focus for a more inclusive public health response to gambling-related harm.

## Conclusion

This review examined how individuals experiencing gambling-related harm are referred to and supported within formal treatment systems, with particular attention to referral pathways, institutional readiness, and treatment engagement. The synthesis of 39 studies reveals a fragmented landscape where referrals are largely self-initiated, treatment entry is inconsistent, and coordination across services remains weak. Despite an increasing number of RG tools, there is little evidence of formalized pathways connecting gambling environments to healthcare systems. As a result, the burden of recognizing harm and initiating help-seeking falls disproportionately on the individuals, reinforcing a crisis-driven approach to gambling-related mental healthcare.

Addressing these gaps in care requires structural reform. Key priorities include developing integrated referral systems, strengthening cross-sector accountability, and embedding public health perspectives within both regulatory and clinical domains. This shift involves moving beyond isolated, individual-focused interventions toward coordinated system-level approaches that recognize gambling-related harm as a shared societal and institutional responsibility. To bridge current system divides, research and policy should prioritize implementation models that clearly establish operational handoff protocols, referral tracking mechanisms, and coordinated linkages between digital gambling platforms and formal healthcare services. Formalizing such referral infrastructures would support a transition from reactive, crisis-driven responses toward earlier intervention aligned with a public health framework approach.

## Data Availability

The original contributions presented in the study are included in the article/Supplementary material, further inquiries can be directed to the corresponding author.
