# Supplementary material for: Structural gaps in referral and treatment pathways for gambling-related harm: a systematic review of health system responses using the antecedents–decision–outcomes framework
Source: Front Public Health. 2026 Jun 19;14:1823843. doi: 10.3389/fpubh.2026.1823843 (PMC13328000; doi:10.3389/fpubh.2026.1823843)
Supplement: Supplementary file 1 [file Data_Sheet_1.pdf]

Appendix I

Documentation of search strategies
University Library search consultation group

Topic/research question: Policies and strategies of gambling intervention – current status and future research agenda within both healthcare and industry.

Name of researcher(s):

Nathan Lakew, Department of Clinical Neuroscience, KI
Philip Lindner, Department of Clinical Neuroscience, KI

Librarian(s): Ingrid Andersson and Emma-Lotta Säätelä, University Library, KI

Databases:

1. Medline (Ovid)
2. Web of Science (Clarivate)
3. PsycInfo (EBSCO)

Total number of hits:

- Before deduplication: 8,178
- After deduplication: 5,235

Karolinska Institutet

1. Medline

Interface: Ovid MEDLINE(R) ALL<br><br>Date of Search: May 30, 2024<br><br>Number of hits: 2,277<br><br>Comment: In Ovid, two or more words are automatically searched as phrases; i.e. no quotation marks are needed	Field labels<br><br>• exp/ = exploded MeSH term<br>• / = non exploded MeSH term<br>• .ti,ab,kf. = title, abstract and author keywords<br>• adjx = within x words, regardless of order<br>• * = truncation of word for alternate endings

Database(s): **Ovid MEDLINE(R) ALL** 1946 to May 29, 2024

**No.**	**Searches**	**Results**
1	Gambling/	7 148
2	gambl*.ti,ab,kf.	12 286
3	1 or 2	13 068
4	exp Health Services/	2 484 414
5	exp Public Health Practice/	847 096
6	Primary Health Care/	94 300
7	Secondary Care/	1 033
8	(((primary or secondary or special* or public or mental or preventive* or communit*) adj3 (care or health or medicine*)) or health care* or healthcare* or health service* or welfare or rehabilit*).ti,ab,kf.	1 873 181
9	4 or 5 or 6 or 7 or 8	4 283 534
10	exp Industry/	359 797
11	exp Commerce/	71 409
12	(industr* or commerc* or company* or companies* or busine* or organisati* or organizati* or operator* or govern* or management* or board* or corporat* or regulat* or provider*).ti,ab,kf.	5 526 295
13	10 or 11 or 12	5 807 109
14	9 or 13	9 026 423
15	3 and 14	3 808
**16**	**limit 15 to yr="2014 -Current"**	**2 277**

Karolinska Institutet

2. Web of Science Core Collection

Interface: Clarivate Analytics<br><br>Editions = A&HCI , ESCI , SCI-EXPANDED , SSCI<br><br>Date of Search: May 30, 2024<br><br>Number of hits: 3,997	Field labels<br><br>• TS/Topic = title, abstract, author keywords and Keywords Plus<br>• NEAR/x = within x words, regardless of order<br>• * = truncation of word for alternate endings<br><br>Note: the *Exact search*-function was used for all the searches

No.	Searches	Results
1	TS=(gambl*)	22 600
2	TS=((((primary OR secondary OR special* OR public OR mental OR preventive* OR community) NEAR/3 (care OR health OR medicine*)) OR "health care*" OR healthcare* OR "health service*" OR welfare OR rehabilit*))	2 095 854
3	TS=((industr* OR commerc* OR company* OR companies* OR busine* OR organisati* OR organizati* OR operator* OR govern* OR management* OR board* OR corporat* OR regulat* OR provider*))	10 398 698
4	#2 OR #3	11 790 209
5	#1 AND #4	5 870
**6**	**#5 Timespan: 2014-01-01 to 2024-12-31**	**3 997**

3. Psycinfo

Interface: EBSCO<br><br>Date of Search: May 30, 2024<br><br>Number of hits: 1,904	Field labels<br><br>• DE = subject heading<br>• TI = title<br>• AB = abstract<br>• KW = author keywords<br>• Nx = within x words, regardless of order<br>• * = truncation of word for alternate endings<br><br>Note: sometimes "quotation marks" are needed for single search terms to avoid automatic term mapping (lemmatization)

No.	Searches	Results
S01	(DE "Gambling") OR (DE "Gambling Disorder")	10 360
S02	TI gambl* OR AB gambl* OR KW gambl*	14 512
S03	((((DE "Health Care Services" OR DE "Behavioral Health Services" OR DE "Continuum of Care" OR DE "Gender Affirming Care" OR DE "Health Care Delivery" OR DE "Health Screening" OR DE "Hospice" OR DE "Hospital Programs" OR DE "Long Term Care" OR DE "Mental Health Services" OR DE "Palliative Care" OR DE "Patient Centered Care" OR DE "Primary Health Care" OR DE "Reproductive Health Care" OR DE "Social Prescribing" OR DE "Spiritual Care") OR (DE "Community Services" OR DE "Community Mental Health Services" OR DE "Community Welfare Services" OR DE "Emergency Services" OR DE "Home Care" OR DE "Home Visiting Programs" OR DE "Public Health Services")) OR (DE "Social Services" OR DE "Adult Day Care" OR DE "Community Services" OR DE "Elder Care" OR DE "Family Preservation" OR DE "Family Reunification" OR DE "Foster Care" OR DE "Government Programs" OR DE "Independent Living Programs" OR DE "Outreach Programs" OR DE "Protective Services" OR DE "Social Casework" OR DE "Social Programs" OR DE "Support Groups")) OR (DE "Electronic Health Services" OR DE "Digital Interventions" OR DE "Health Information Technology" OR DE "Precision Medicine")) OR (DE "Treatment" OR DE "Addiction Treatment" OR DE "Adjunctive Treatment" OR DE "Aftercare" OR DE "Alternative Medicine" OR DE "Anxiety Management" OR DE "Behavior Therapy" OR DE "Bibliotherapy" OR DE "Brief Interventions" OR DE "Caregiving" OR DE "Client Transfer" OR DE "Client Treatment Matching" OR DE "Computer Assisted Therapy" OR DE "Conversion Therapy" OR DE "Counseling" OR DE "Creative Arts Therapy" OR DE "Cross Cultural Treatment" OR DE "Culturally Adapted Interventions" OR DE "Electronic Health Services" OR DE "Exercise Therapy" OR DE "Habilitation" OR DE "Health Care Services" OR DE "Human Potential Movement" OR DE "Human Services" OR DE "Institutionalization" OR DE "Integrated Services" OR DE "Interdisciplinary Treatment Approach" OR DE "Intervention" OR DE "Involuntary Treatment" OR DE "Life Sustaining Treatment" OR DE "Maintenance Therapy" OR DE "Medical Treatment (General)" OR DE "Mentalization-Based Interventions" OR DE "Milieu Therapy" OR DE "Mind Body Therapy" OR DE "Mindfulness-Based Interventions" OR DE "Movement Therapy" OR DE "Multimodal Treatment Approach" OR DE "Multisystemic Therapy" OR DE "Nature-Based Interventions" OR DE "Outpatient Treatment" OR DE "Pain Management" OR DE "Physical Treatment Methods" OR DE "Private Practice" OR DE "Psychoeducation" OR DE "Psychosocial Interventions" OR DE "Psychotherapy" OR DE "Recreation Therapy" OR DE "Rehabilitation" OR DE "Respite Care" OR DE "Self-Help Techniques" OR DE "Sex Therapy" OR DE "Social Services" OR DE "Sociotherapy" OR DE "Strengths-Based Interventions" OR DE "Stress Management" OR DE "Symptoms Based Treatment" OR	688 464

	DE "Therapeutic Processes" OR DE "Transdiagnostic Treatment" OR DE "Trauma-Informed Care" OR DE "Trauma Treatment" OR DE "Treatment Guidelines" OR DE "Treatment Outcomes" OR DE "Treatment Planning" OR DE "Video-Based Interventions" OR DE "Preventive Health Services" OR DE "Preventive Mental Health Services" OR DE "Prophylactic Drug Therapy")	
S04	TI ((((primary OR secondary OR special* OR public OR mental OR preventive* OR community) N3 (care OR health OR medicine*)) OR "health care*" OR healthcare* OR "health service*" OR welfare OR rehabilit*)) OR AB ((((primary OR secondary OR special* OR public OR mental OR preventive* OR community) N3 (care OR health OR medicine*)) OR "health care*" OR healthcare* OR "health service*" OR welfare OR rehabilit*)) OR KW ((((primary OR secondary OR special* OR public OR mental OR preventive* OR community) N3 (care OR health OR medicine*)) OR "health care*" OR healthcare* OR "health service*" OR welfare OR rehabilit*))	618 534
S05	DE "Business" OR DE "Business Ethics" OR DE "Business Investments" OR DE "Commerce" OR DE "Commercialization" OR DE "Entrepreneurship" OR DE "Family Business" OR DE "Hospitality Industry" OR DE "Ownership" OR DE "Pharmaceutical Industry" OR DE "Public Relations" OR DE "Research and Development" OR DE "Supply Chain Management" OR DE "Supply Chains" OR DE "Tourism" OR DE "Business Organizations" OR DE "Family Business" OR DE "Multinational Corporations" OR DE "Small Businesses" OR DE "Organizational Behavior" OR DE "Organizational Objectives" OR DE "Corporate Social Responsibility" OR DE "Employee Interaction" OR DE "Organizational Citizenship Behavior" OR DE "Organizational Effectiveness" OR DE "Organizational Politics"	139 431
S06	TI ((industr* OR commerc* OR company* OR companies* OR busine* OR organisati* OR organizati* OR operator* OR govern* OR management* OR board* OR corporat* OR regulat* OR provider*)) OR AB ((industr* OR commerc* OR company* OR companies* OR busine* OR organisati* OR organizati* OR operator* OR govern* OR management* OR board* OR corporat* OR regulat* OR provider*)) AND KW ((industr* OR commerc* OR company* OR companies* OR busine* OR organisati* OR organizati* OR operator* OR govern* OR management* OR board* OR corporat* OR regulat* OR provider*))	361 774
S07	S3 OR S4	1 051 301
S08	S5 OR S6	416 952
S09	S7 OR S8	1 380 694
S10	S1 OR S2	15 237
S11	S9 AND S10	3 727
**S12**	**S9 AND S10 Limiters - Publication Year: 2014-2024**	**1 904**
