# Supplementary material for: Structural gaps in referral and treatment pathways for gambling-related harm: a systematic review of health system responses using the antecedents–decision–outcomes framework
Source: Front Public Health. 2026 Jun 19;14:1823843. doi: 10.3389/fpubh.2026.1823843 (PMC13328000; doi:10.3389/fpubh.2026.1823843)
Supplement: Supplementary file 2 [file Data_Sheet_2.pdf]

# Appendix II

**Table of Contents** **Page**

| | |
|---|---|
| Supplementary Table 1. Summary of studies included in the review | 2 |
| Supplementary Table 2: Covidence data extraction sheet template | 6 |
| Supplementary Figure 1: Overview *of the systematic review process and data analysis methodology* | 3 |
| Supplementary Figure 4: *Co-word network of term clusters based on 39 included studies (2014–2024) analyzing platform-based gambling harm* | 4 |
| Supplementary Figure 3: *TF-IDF weighted heatmap showing contextually salient relationships between key abstract terms* | 4 |
| Supplementary Table 3: Manifest content analysis of the included articles | 5 |
| Figure 6. NVivo-coded project maps displaying the thematic hierarchy | 8 |

Supplementary Table 1. Summary of studies included in the review

| Title | Year | Author(s) | Journal name | Study design | Research theme/findings |
|---|---|---|---|---|---|
| A six-step brief intervention to reduce distress and increase treatment readiness in problem gamblers | 2020 | Oakes et al. | Australasian Psychiatry | Mixed research | Presents a structured six-step intervention to stabilize gamblers in an emotional crisis, using the moment of contact to increase readiness and guide users toward further treatment options. |
| A systematic review of treatments for problem gambling | 2017 | Nancy M. et al. | Psychology of Addictive Behaviors | Systematic review | Synthesizes results from 22 RCTs, concluding that CBT with some therapist involvement is the most promising approach, though structural and methodological inconsistencies persist across studies. |
| Acceptability of Internet-based interventions for problem gambling: a qualitative study of focus groups with clients and clinicians | 2019 | Sanchez et al. | BMC Medical Informatics and Decision Making | Qualitative research | Explores acceptability of therapist-guided IBIs for problem gambling, emphasizing that client and clinician buy-in depends on emotional safety, structural integration, and usability. |
| Age of pathological gambling onset: Clinical and treatment-related features | 2014 | Shin et al. | Journal of Addiction Medicine | Mixed research | Age of PG onset influences treatment delays, pharmacological response patterns, and gambling profiles, offering guidance for tailoring age-specific interventions. |
| An Examination of Clinician Responses to Problem Gambling in Community Mental Health Services | 2020 | Manning et al. | Journal of Clinical Medicine | Cross-sectional study | Highlights limited PG screening and referral among clinicians despite high case prevalence; points to system-level training and integration gaps. |
| Analyzing Consumer Protection for Gamblers Across Different Online Gambling Operators: A Replication Study | 2023 | Catania & Griffiths | International Journal of Mental Health and Addiction | Descriptive audit study with simulated client interactions | Investigates how online gambling operators implement consumer protection tools and responsible gambling practices, replicating a previous audit. It reveals improvements in RG tool availability but identifies gaps in customer service responses and consistency of help mechanisms. |
| Assessing the Need for Higher Levels of Care Among Problem Gambling Outpatients | 2017 | Ledgerwood & Arfken | Journal of Gambling Studies | Cross-sectional study | Outpatient clients and clinicians identify structural unmet needs for residential or IOP services, especially for those with comorbidity and high gambling severity. |
| Association of sociodemographic, psychopathological, and gambling-related factors with treatment utilization for pathological gambling | 2014 | Bischof et al. | European Addiction Research | Mixed research | Severity, social pressure, and negative consequences predict treatment use as well as comorbidity or demographics. |
| Barriers and Facilitators of Responding to Problem Gambling: Perspectives from Australian Mental Health Services | 2017 | Rodda et al. | Journal of Gambling Studies | Qualitative research | Mental health clinicians rarely screen for gambling harm due to prioritization, lack of tools, and structural system gaps, but are receptive to mandated screening and training. |
| Barriers in Access to the Treatment for People with Gambling Disorders. Are They Different from Those Experienced | 2016 | Dąbrowska et al. | Journal of Gambling Studies | Qualitative research | Gambling-specific treatment access is hindered by a dual burden of stigma and inadequate system adaptation, with barriers both similar to and distinct from those in substance use treatment. |

| | | | | | |
|---|---|---|---|---|---|
| by People with Alcohol and/or Drug Dependence? | | | | | |
| Beyond Reno: A Critical Commentary on Hancock and Smith | 2017 | Abbott | International Journal of Mental Health Addiction | Text and opinion | Argues that individual-responsibility models (like the Reno Model) have stifled harm reduction and endorses a shift to a consumer protection–oriented public health approach. |
| Brief Intervention Within Primary Care for At-Risk Gambling: A Pilot Study | 2016 | Nehlin et al. | Journal of Gambling Studies | Mixed research | BI is feasible in primary care for at-risk gambling but requires familiar caregiver delivery, better tools, and strong engagement to ensure participation. |
| Brief telephone interventions for problem gambling: a randomized controlled trial | 2018 | Abbott et al. | Addiction | Randomized controlled trial | Large-scale trials found that brief telephone-based interventions, including MI and self-help enhancements, yield durable gambling reductions, particularly for participants with higher psychological distress and gambling severity. |
| Characteristics and help-seeking behaviors of Internet gamblers based on most problematic mode of gambling | 2015 | Hing et al. | Journal of Medical Internet Research | Cross-sectional study | Internet problem gamblers were less likely to seek help than land-based gamblers, despite lower distress and problem severity; both showed limited uptake of online help services. |
| Characteristics of Gamblers Who Use the French National Problem Gambling Helpline and Real-Time Chat Facility: Longitudinal Observational Study | 2020 | Darbeda et al. | JMIR Formative Research | Quantitative, multivariate regression analysis | Gamblers who used the service were demographically diverse; referral was influenced by gambling type, severity, and service access channel, with online young users less likely to be referred to formal treatment. |
| Core Competencies for Disordered Gambling Counsellors: A Modified Delphi Study | 2020 | McDowell et al. | JMIR Formative Research | Qualitative research | Identifies a comprehensive set of expert-validated core competencies that define professional readiness for gambling treatment providers, intended to inform training, supervision, and care quality. |
| Critiquing the Reno Model I-IV International Influence on Regulators and Governments (2004-2015)- the Distorted Reality of "Responsible Gambling" | 2017 | Hancock & Smith | International Journal of Mental Health and Addiction | Conceptual and critical analysis | Argues that the Reno Model has legitimized ineffective, industry-friendly interpretations of responsible gambling that deflect responsibility from gambling systems and regulators to individual gamblers, weakening public health protections. |
| Do Online Gambling Products Require Traditional Therapy for Gambling Disorder to Change? Evidence from Focus Group Interviews with Mental Health Professionals Treating Online Gamblers | 2021 | Lopez-Gonzalez et al. | Journal of Gambling Studies | Qualitative research | Mental health professionals identify that current gambling treatments inadequately address the needs of online gamblers, particularly due to their younger age, tech dependence, resistance to abstinence, and the unique structures of skill-based online gambling. |
| Early detection of pathological gambling: betting on GPs' beliefs and attitudes | 2014 | Achab et al. | BioMed Research International | Cross-sectional study | Although most GPs recognize gambling as a significant public health concern, few systematically screen for it, and many lack knowledge of referral networks, highlighting a major gap in early detection and treatment channelization within primary care. |

| | | | | | |
|---|---|---|---|---|---|
| Exploring the feasibility of a gambling harm screening model in general practice and community service settings in Fairfield: a pilot study | 2024 | Reid et al. | Australian Journal of Primary Health | Qualitative research | Evaluates a co-designed gambling screening model in GP and community settings, implementation barriers and opportunities among communities. |
| Filling the GAP: Integrating a gambling addiction program into a shelter setting for people experiencing poverty and homelessness | 2022 | Matheson et al. | PLOS ONE | Qualitative research | Evaluates an integrated, shelter-based gambling addiction program tailored to address the compounded challenges of problem gambling, poverty, and homelessness |
| Gamblers Anonymous as a Recovery Pathway: A Scoping Review | 2016 | Schuler et al. | Journal of Gambling Studies | Scoping review | Synthesizes evidence on GA's effectiveness, cultural framing, and implementation issues with mixed outcomes and significant gaps in long-term effectiveness research |
| Gambling Disorder Treatment Referrals Within the Irish Mental Health Service: A National Survey Using Freedom of Information Requests | 2018 | Columb et al. | International Journal of Mental Health and Addiction | Descriptive survey (mapping) | Identifies major inconsistencies and gaps in how gambling disorder referrals are processed and treated across Ireland's public mental health infrastructure. |
| Gambling treatment service providers' views about contingency management: a thematic analysis | 2022 | Dorey et al. | Harm Reduction Journal | Qualitative research | Explores practitioner attitudes, ethical concerns, and implementation barriers related to the use of CM (incentive-based approaches) in gambling treatment. |
| Longitudinal Assessment of a Manualized Group Treatment Program for Gambling Disorder: The Ohio Problem Gambling Treatment Model for Adults with Co-Occurring Disorders | 2021 | Kruse-Diehr et al. | Journal of Gambling Studies | Mixed research | Assess the effectiveness of a manualized gambling treatment model targeting individuals with co-occurring mental health and substance use disorders. |
| Policies and interventions to reduce harmful gambling: an international Delphi consensus and implementation rating study | 2022 | Regan et al. | The Lancet Public Health | Qualitative research | Establishes consensus on 81 effective policy measures across seven domains of gambling harm prevention and rates 40 for implementation feasibility. |
| Predicting Online Problem Gambling Treatment Discontinuation: New Evidence from Cross-Validated Models | 2023 | Palomäki et al. | Psychology of Addictive Behaviors | Predictive modeling using a real-world program | Identifies key predictors of discontinuation from a national online treatment program and suggests design improvements to support retention. |
| Proposing a health promotion framework to address gambling problems in Australian Indigenous communities | 2018 | Fogarty et al. | Health Promotion International | Case report | Discusses the adoption of culturally grounded, health promotion–oriented frameworks to address gambling harms in Indigenous communities. |
| Should screening for risk of gambling-related harm be undertaken in health, care and support settings? A systematic review of the international evidence | 2021 | Blank et al. | Addiction Science & Clinical Practice | Systematic review | This review evaluates the feasibility and early evidence base for implementing screening and brief intervention (SBIRT) for gambling-related harm in diverse support settings. |

| | | | | | |
|---|---|---|---|---|---|
| Single-session interventions for problem gambling may be as effective as longer treatments: Results of a randomized control trial | 2016 | Toneatto | Addictive Behaviors | Randomized controlled trial | Evaluates the comparative effectiveness of brief vs. multi-session therapies for gambling disorder; suggests minimal interventions may suffice for some clients. |
| Subtyping based on readiness and confidence: the identification of help-seeking profiles for gamblers accessing web-based counselling | 2015 | Rodda et al. | Addiction | Quantitative subtype analysis with multinomial regression | Profiles gamblers by motivational subtypes based on importance, readiness, and confidence to change, identifying implications for tailoring interventions in web-based settings. |
| The diagnostic accuracy of brief screening instruments for problem gambling: A systematic review and meta-analysis | 2019 | Dowling et al. | Clinical Psychology Review | Systematic review | Provides a comprehensive comparison of brief gambling screening tools and their validity across various settings, populations, and timeframes. |
| The efficacy of three modalities of Internet-based psychotherapy for non-treatment-seeking online problem gamblers: A randomized controlled trial | 2016 | Luquiens et al. | Journal of Medical Internet Research | Randomized controlled trial | Assesses the efficacy and acceptability of online psychotherapy modalities (CBT, feedback, guidance) among at-risk poker players not actively seeking help. |
| The General Population's View on Where to Seek Treatment for Gambling Disorder - a General Population Survey | 2019 | Håkansson & Ford | Psychology Research and Behavior Management | Cross-sectional study | Investigates public beliefs about whether gambling disorder warrants professional treatment and which institutions are seen as suitable. |
| The Typologies of Mental Health, Addiction, and Problem Gambling Systems Integration in Ontario | 2022 | Mfoafo-M'Carthy et al. | International Journal of Mental Health and Addiction | Qualitative research | Explores how problem gambling services are integrated (or sidelined) within broader mental health and addiction systems in Ontario, highlighting uneven implementation and structural neglect. |
| Treatment for problem gambling and counselors' perception of their clinical competence: a national web survey in Sweden | 2022 | Månsson et al. | Addiction Science & Clinical Practice | Cross-sectional study | Maps the current treatment provision and change technique prioritization among Swedish counselors, and explores predictors of their self-perceived competence |
| Treatment-seeking precipitators in problem gambling: analysis of data from a gambling helpline | 2014 | Valdivia-Salas et al. | Psychology of Addictive Behaviors | Quantitative research | Identifies key factors (precipitators and helpline operator differences) that predict whether gamblers follow through on treatment referrals after calling a helpline. |
| Web-Based Intervention and Email-Counseling for Problem Gamblers: Results of a Randomized Controlled Trial | 2020 | Jonas et al. | Journal of Gambling Studies | Randomized controlled trial | Evaluates engagement, outcomes, and therapeutic experience across two modes of digital intervention targeting non-treatment-seeking problem gamblers |
| What mental health professionals in Israel know and think about adolescent problem gambling | 2015 | Sansanwal et al. | International Gambling Studies | Cross-sectional study | Assesses knowledge, attitudes, training, and referral confidence regarding adolescent problem gambling among professionals. |

Supplementary Table 2. Covidence data extraction sheet template

| Categories | Definition | Category field type |
|---|---|---|
| **Manuscript meta information** | | |
| Year | Year of publication | Text field |
| Author(s) | Manuscript author(s) | Text field |
| Title | Title of the manuscript | Text field |
| Study design | Methodology used/sample description/review type | Text field |
| Country | Country in which the study conducted | Text field and Check box (US, UK, Cananda, Australia, others) |
| Journal name | Where the publication appears | Text field |
| **Characteristics of the study** | | |
| Method description | Methodology used/sample description/review type | Text field |
| Study design | Research method | Text filed and check box (e.g., RCT, Cross sectional, . . ., Other) |
| Context of the study | Research setting | Text field (e.g., Helpline, Primary care, . . . ) |
| Themes/Findings | Objective and main research findings | Text field |
| **ADO summary** | | |
| ADO Summary | Which ADO construct(s) is the focus in the article | Check box (Antecedents, Decision, Outcome) |
| ADO notes | How the ADO constructs are discussed | Text field |
| **Pathway to treatment** | | |
| Treatment system type | What treatment system type was used | Check box (e.g., public health, crisis support, private clinics, online therapy, self-help) |
| Referral type | How the patient was referred to formal treatment care | Check box (e.g., self-initiated, operator triggered, Health professional referral, peer/family referral, Helpline) |
| Framing responsibility | How the paper frames pathway to treatment | Check box (e.g., public health framing, system responsibility, individual responsibility, operators responsibility, blended responsibility) |
| Operators channelization | Whether or operators is involved in the referral to formal treatment process | Boolean field (Yes/No) |
| Quality/description | General notes | Text field |

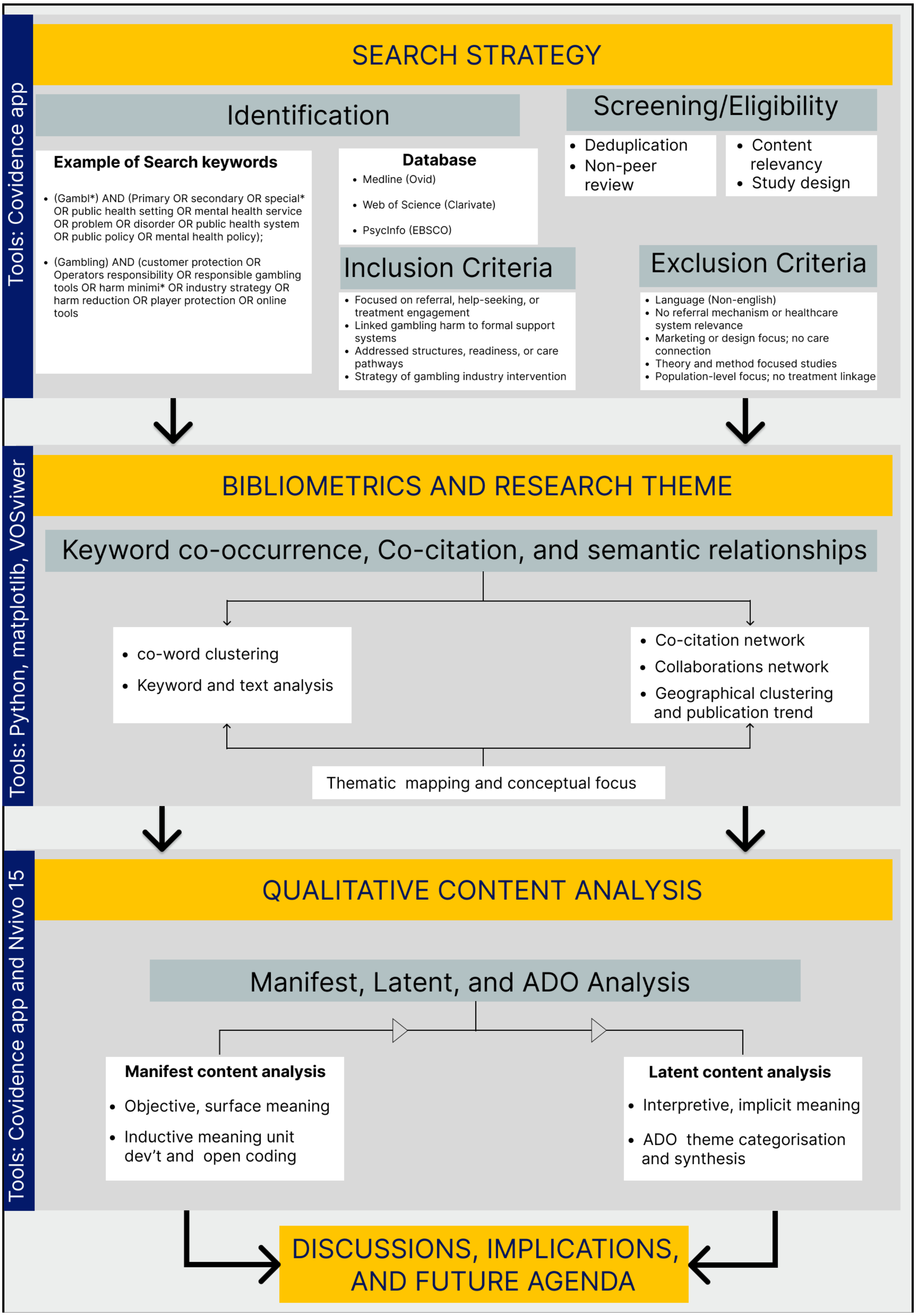


Supplementary Figure 1. *Overview of the systematic review process and data analysis methodology*

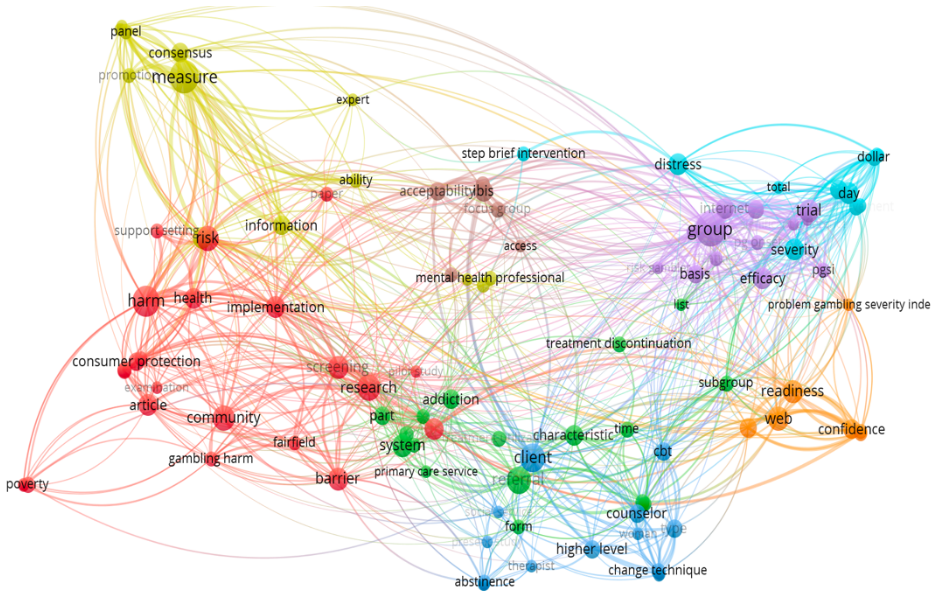


Supplementary Figure 2. Co-word network of term clusters based on 39 included studies (2014–2024) analyzing platform-based gambling harm

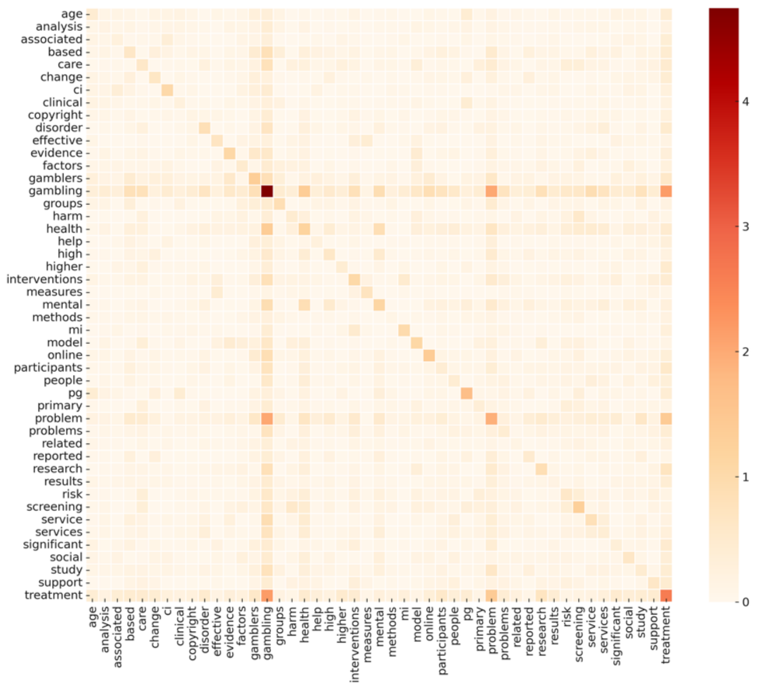


Supplementary Figure 3: TF-IDF weighted heatmap showing contextually salient relationships between key abstract terms

Supplementary Table 4: Manifest content analysis of the included articles

| Citation | Context of study | ADO construct(s) | ADO notes |
|---|---|---|---|
| Oakes et al. (2020) | Helpline setting | Decision; Outcome | Decision: Help-seeking trigger, helpline-based intervention design<br>Outcome: Referral success, emotional stabilization, treatment readiness |
| Nancy et al. (2017) | Multisite trial reviews | Decision; Outcome | Outcomes: Reported outcomes, treatment engagement, linkage success/failure<br>Decision: Intervention choice and design, help-seeking |
| Sanchez et al. (2019) | Healthcare system | Antecedents | Barriers to treatment and determinants of acceptability before IBI implementation |
| Shin et al. (2014) | Specialized PG outpatient clinic | Outcome | Treatment patterns, response predictors, medication use, and help-seeking delay analyzed by age of PG onset |
| Manning et al. (2020) | Community mental health settings | Antecedents; Decision | ADO Antecedents: System readiness, service visibility, operator readiness<br>Decision: Clinician screening, referral decision |
| Catania & Griffiths (2023) | Gambling Platforms | Antecedents | Operator and System Readiness (not applied to referral) |
| Ledgerwood & Arfken (2017) | Healthcare (community-based outpatient clinics) | Antecedents | System Readiness, Personal Barriers, Recognition & Awareness |
| Bischof et al. (2014) | Multi-source community and clinical population sample | Antecedents | Personal Barriers, Recognition & Awareness, Framing Responsibility |
| Rodda et al. (2017) | Mental health services (community, private, public, crisis, and support setting) | Antecedents | Identifies key barriers to routine gambling screening in mental health care, including competing priorities, tool unavailability, training deficits, and stigma concerns. |
| Dąbrowska et al. (2016) | Public treatment system, social welfare, psychiatric and addiction services | Antecedents; Decision | Antecedents: structural and institutional barriers such as system mismatch, stigma, poor therapist training, and lack of formal mandates for gambling treatment<br>Decision: delayed help-seeking or redirection to unrelated services |
| Abbott (2017) | Gambling research, policy analysis, and RG discourse | Antecedents | The commentary critiques structural barriers to harm reduction, particularly industry and state interests, and highlights alternative national models for system-level reform. |
| Nehlin et al. (2016) | Primary care health centers | Decision | Identifies conditions for successful BI delivery during health visits, emphasizing timing, caregiver relationship, and screening ease. |
| Abbott et al. (2018) | National gambling helpline | Outcome | Structured interventions support long-term reductions in gambling behavior, financial loss, and improved goal achievement. |
| Hing et al. (2015) | National online gambling and help-seeking sample | Antecedents | Help-seeking varies by modality, demographics, severity, and promotion exposure; internet gamblers show lower uptake despite available tools. |
| Darbeda et al. (2020) | Longitudinal observational analysis of 9,474 helpline/chat contacts, with | Antecedents; Decision; Outcome | The study identifies behavioral, structural, and modality-specific factors that predict referral to treatment, decisions, and success rate. It highlights |

| | | | |
|---|---|---|---|
| | multivariate model of referral likelihood | | demographics, gambling type, and severity indicators are to likely influences on service uptake. |
| McDowell et al. (2020) | Multinational expert panel evaluation of gambling treatment competencies | Antecedents | Highlights structural and professional prerequisites for effective counselling, including core knowledge, techniques, and cultural competence. |
| Hancock & Smith (2017) | Global/regulatory, with focus on influence of the Reno Model on gambling policy | Antecedents | Examines how industry framing positioned gambling harm as an individual issue, downplaying system accountability and hindering systemic reform. |
| Lopez-Gonzalez et al. (2022) | Public and NGO-based gambling treatment organizations in Spain | Antecedents; Decision | Antecedents: Systemic mismatch between traditional CBT models and online gamblers' profiles; providers face uncertainty adapting to new modalities.<br>Decision: Therapists adjust goals and techniques to address low motivation and resistance to standard CBT among online gamblers. |
| Achab et al. (2014) | Primary care settings across six Swiss cantons | Antecedents | Highlights the lack of routine screening, poor knowledge of treatment systems, and low confidence in addressing problem gambling in primary care. |
| Reid et al. (2024) | Primary care and community health services | Antecedents; Decision; Outcome | System level readiness, community stigma, evaluation of staff implementation, and outcome (e.g., referral follow-up) |
| Matheson et al. (2022) | Multi-service shelter-based agency in Toronto applying a community-based harm-reduction approach | Antecedents; Decision; Outcome | High rates of undetected gambling harm needs were met with fragmented service responses. A structured but flexible, client-centered approach was used to engage individuals through group and one-on-one support. Reported outcomes included reduced gambling, financial and housing stabilization, and improved self-agency. |
| Schuler et al. (2016) | Review with a focus on GA as a mutual aid recovery model for problem gambling | Decision; Outcome | Decision: individuals engage with GA as a help-seeking pathway, including motivations, perceived fit, and stigma barriers.<br>Outcomes: Synthesizes evidence on abstinence, relapse prevention, and coping |
| Columb et al. (2018) | National mental health system and regional drug task forces in Ireland | Antecedents; Outcome | Antecedents: Systemic fragmentation, lack of standardized treatment pathways, and uneven recognition of gambling disorder across the health system<br>Outcomes: Varied referral outcomes (some treatment, some referred elsewhere, others rejected); extremely low formal engagement rates |
| Dorey et al. (2022) | National gambling treatment services, UK | Decision; Outcome | Decision: Focuses on providers' beliefs about whether and how CM can influence treatment engagement (e.g., concerns about motivation and ethical dilemmas.<br>Outcomes: somewhat mixed perceptions of CM's effectiveness and concerns about relapse, manipulation, or erosion of therapeutic trust. |
| Kruse-Diehr et al. (2021) | State-funded public mental health settings | Outcome | Significant reductions in gambling disorder severity among completers; and non-significant changes in gambling urges and self-esteem; highlight the feasibility and partial effectiveness of integrated manualized care for co-occurring mental health populations. |

| | | | |
|---|---|---|---|
| Regan et al. (2022) | System-level policy evaluation framed as a public health blueprint | Antecedents; Outcome | Antecedents: Systemic neglect and policy incoherence, dominance of individual responsibility, lack of integrated treatment infrastructure<br>Outcomes presents 40 prioritized measures likely to reduce harm if implemented, including in treatment and referral systems |
| Palomäki et al. (2023) | Online therapist-guided CBT program, supported by the Finnish National Helpline | Antecedents; Outcome | Antecedents: Key predictors of discontinuation—age, education, readiness to change, social support, waiting list time, gambling severity<br>Outcomes: Actual dropout rates at three treatment stages and emphasizes the need for program design improvements to reduce attrition |
| Fogarty et al. (2018) | Indigenous communities in the Northern Territory, New South Wales, and Western Australia | Antecedents; Decision | Antecedents: Highlights lack of culturally appropriate, coordinated services; stigma and community-level silence on gambling harms<br>Decision: Case studies show how culturally grounded community engagement, art-based education, and peer ambassador models facilitate help-seeking and early action |
| Blank et al. (2021) | Identifies population-level screening interventions for gambling harm across general healthcare, mental health, addiction, and social services. | Antecedents; Decision | Antecedents: System-level unpreparedness for routine gambling screening, weak referral systems, lack of training.<br>Decision: Initial feasibility and acceptability of SBIRT interventions explored across health, mental health, and financial counseling services |
| Toneatto (2016) | Community-recruited participants at the Centre for Addiction and Mental Health, Toronto | Decision; Outcome | Decision: The study explores treatment uptake and modality preference as a key decision point<br>Outcomes: Assesses reduction in gambling frequency, expenditure, DSM-IV criteria, and satisfaction across all four intervention arms |
| Rodda et al. (2015) | National online gambling counselling platform | Antecedents; Decision | Antecedents: Examines how demographic, motivational, and gambling factors shape help-seeking readiness<br>Decision: Provides evidence on motivational readiness subtypes as decision-stage differentiators in a digital help context |
| Dowling et al. (2019) | International, with a focus on general population, primary care, mental health, and AOD services | Antecedents | Emphasizes systemic barriers to screening implementation such as lack of training, low prioritization in care settings, stigma, poor referral pathways, and limited tool awareness |
| Luquiens et al. (2016) | Fully online, industry-facilitated study with passive gambling environment recruitment and self-administered interventions | Antecedents; Decision; Outcome | Antecedents: Non-help-seeking status, low intrinsic motivation, unawareness of support options<br>Decision: Trial participation is influenced by level of effort required by the intervention<br>Outcomes: Minor PGSI reductions in all groups, no significant differences between modalities, and dropout |
| Håkansson & Ford (2019) | General population in Sweden | Antecedents | Public perception of gambling disorder as a medical vs. peer support issue; personal treatment experience and age influence formal treatment preference |
| Mfoafo-M'Carthy et al. (2022) | Community and healthcare treatment systems across Ontario | Antecedents; Decision | Antecedents: Structural integration exists, but PG remains poorly integrated due to underfunding, weak screening tools, and role confusion. |

| | | | |
|---|---|---|---|
| | | | Decision: Referral relies on tools like GAIN-SS, which miss PG cases; team coordination is inconsistent. |
| Månsson et al. (2022) | Sweden's post-2018 public treatment system for problem gambling | Antecedents; Decision | Antecedents: Shows broad heterogeneity in treatment availability, organizational setting, and training; motivation is seen as the most important technique<br>Decision: The decision to apply specific techniques (e.g., CBT, exposure) varies widely; counselor experience correlates with perceived legitimacy and adequacy |
| Valdivia-Salas et al. (2014) | New Mexico Council on Problem Gambling helpline | Antecedents; Decision; Outcome | Financial and family harm were the most predictive motivators for calling and following through with treatment. Operator influence, perceived urgency, and reason for calling predicted treatment uptake. Attendance was highest among those citing financial or family crises, not emotional distress or acute episodes. |
| Jonas et al. (2020) | National online gambling help platform (BZgA) | Antecedents; Decision; Outcome | Self-initiated users of a public website, with low readiness and no prior treatment. Enrollment in a structured program vs. email counseling shows differential engagement and satisfaction patterns. Structured intervention (CO) yielded stronger reductions in gambling severity and improved well-being compared to email or waitlist. |
| Sansanwal et al. (2015) | Mental health and school-based professionals serving youth in Israel | Antecedents | Low perceived seriousness of gambling compared to other adolescent risks; limited training and confidence; minimal school-level resources and policy awareness |

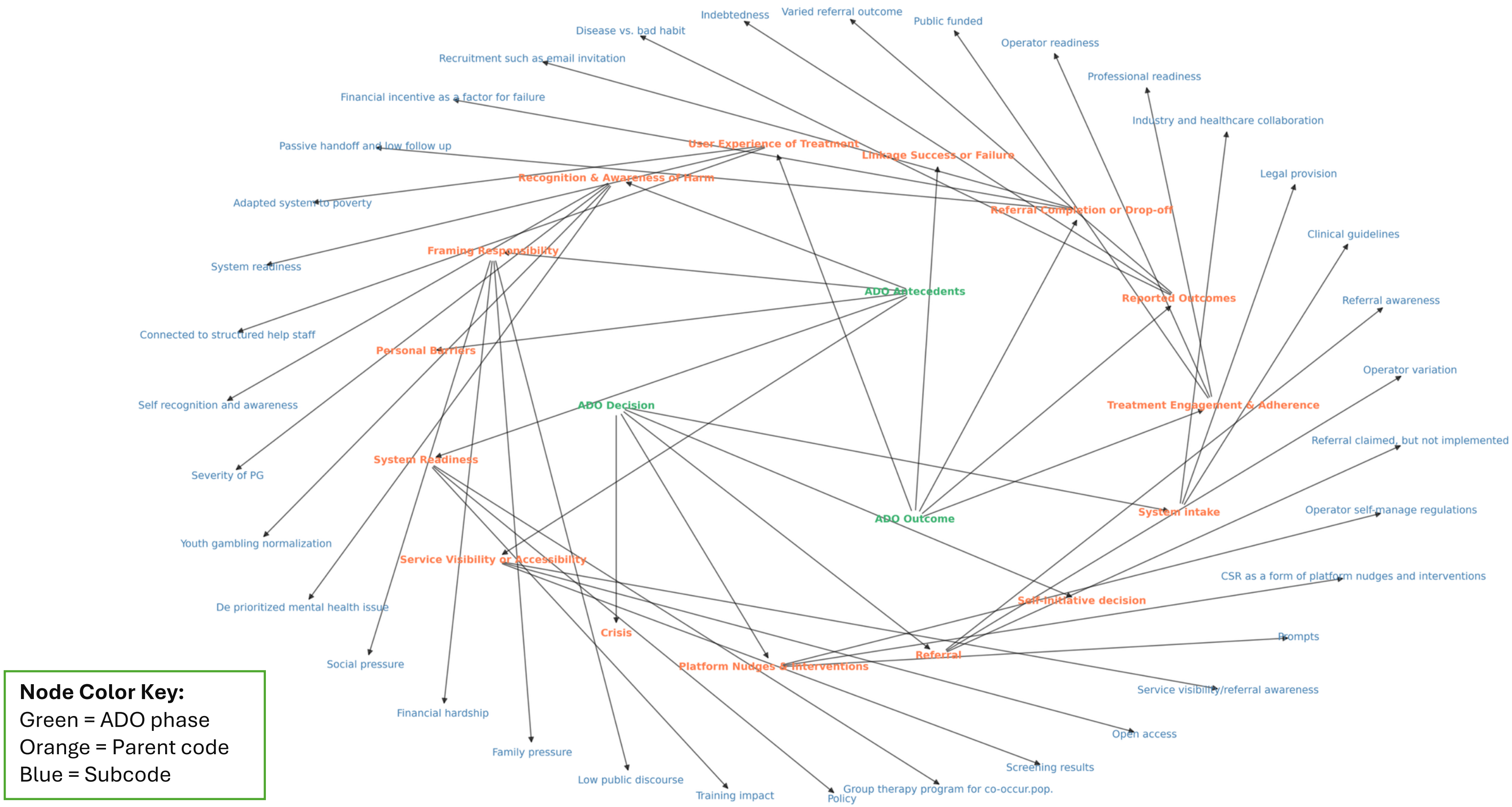


*Figure 6. NVivo-coded project map displaying the thematic hierarchy developed under the ADO framework. The map visualizes phase-level categories, parent themes, and subcodes derived from the 39 full-text analysis ADO based themes.*
