# Supplementary material for: Structural gaps in referral and treatment pathways for gambling-related harm: a systematic review of health system responses using the antecedents–decision–outcomes framework
Source: Front Public Health. 2026 Jun 19;14:1823843. doi: 10.3389/fpubh.2026.1823843 (PMC13328000; doi:10.3389/fpubh.2026.1823843)
Supplement: Supplementary file 3 [file Data_Sheet_3.pdf]

Appendix III: Rapid Quality Appraisal Summary

Study_id	Assessment + Reasoning: <u>Clarity of referral or pathway definition</u>	Assessment + Reasoning: <u>Adequacy of outcome measurement</u>	Assessment + Reasoning: <u>Reporting of attrition or follow-up</u>	Assessment + reasoning: <u>Transparency of data source and sampling</u>	Overall assessment
Darbeda et al. 2020	HIGH -> Referral Type: Helpline / Chat -> Treatment.	HIGH -> Uses measurable outcomes—referral likelihood, referral completion, and demographic predictors—reported quantitatively.	HIGH -> Longitudinal design includes follow-up indicators and repeat-contact analysis, showing changes in engagement over time.	HIGH -> FOI data sources and regional response rates are clearly reported with a transparent mapping method.	Strong system-level mapping and repeat-contact analysis, though follow-up was limited to helpline use only.
Reid et al. 2024	HIGH -> Referral Type: Primary care to community services.	HIGH -> Qualitative outcome indicators reported (staff feedback, referral completion, community response).	HIGH -> Reports post-referral outcomes and follow-up interviews to assess sustainability and acceptability.	HIGH -> Provides a clear contextual description of communities involved, intervention types, and the participatory development process.	Contextually rich case study to illustrate culturally appropriate, community-based strategies. While the intervention piloted structured referral workflows, formal outcome tracking, and system-level integration were not yet in place.
Valdivia-Salas 2014	HIGH -> Referral Type: Helpline to treatment	HIGH -> Quantitative analysis of predictors of treatment attendance and non-attendance, including precipitating factors and operator-level effects.	HIGH -> Referral follow-up tracked through treatment attendance data, enabling measurement of referral completion.	HIGH -> RCT methods, sample size, recruitment criteria, and helpline context are clearly described.	Strong design and transparent reporting; focuses on post-contact support rather than broader system-level referral structures.
Jonas et al. 2020	LOW -> Referral Type: Self-initiated online help -> Digital intervention.	HIGH -> Reports validated measures, including changes in gambling severity, well-being, and engagement duration.	HIGH -> Attrition rates and follow-up assessments are explicitly reported, with differential dropout between groups analyzed.	HIGH -> Clear reporting of sample characteristics, variable definitions, statistical approach, and modeling results.	Sound quantitative study on motivational readiness; statistically transparent but lacks longitudinal or referral pathway analysis.
Kruse-Diehr et al. 2022	LOW -> Referral Type: Public mental-health intake -> Structured group treatment.	HIGH -> Uses validated quantitative measures (gambling severity, urges, self-esteem) and qualitative feedback to assess treatment impact.	HIGH -> Longitudinal assessment includes pre-post comparisons and follow-up evaluation, with attrition discussed.	HIGH -> Detailed Delphi procedures, expert composition, consensus process, and rating methods reported transparently.	Rigorous Delphi study on policy and treatment priorities; methodologically clear but lacks empirical referral data or outcome tracking.
Luquiens et al. 2016	LOW -> Referral Type: Self-enrolled online intervention (no referral process).	HIGH -> Quantitative PGSI and behavioral outcomes were reported across all intervention arms with clear comparisons.	HIGH -> Attrition rates are explicitly reported and analyzed, with dropout patterns discussed in relation to user engagement and intervention burden.	HIGH -> Delphi procedures, panel composition, consensus criteria, and iterative rounds with methodological rigor.	Rigorous Delphi study on structural competencies; informative for system readiness but not focused on referral pathways or clinical outcomes.
Palomäki et al. 2023	LOW -> Referral Type: Helpline-supported online treatment.	HIGH -> Reports quantifiable dropout rates, predictive accuracy metrics, and risk factor profiles across treatment stages.	HIGH -> Attrition explicitly modeled as a study outcome; multi-stage dropout tracked with follow-up indicators.	HIGH -> clearly reports survey methodology, sampling, instruments, and statistical analyses.	Transparent and methodologically sound cross-sectional analysis demonstrating modality differences in help-seeking; does not operationalize referral pathways or examine formal treatment linkage.

Petry & Rash 2017	LOW -> Referral Type: Treatment evaluation (no referral component).	HIGH -> Reports standardized outcomes (gambling severity, treatment engagement, relapse rates) across studies, with comparative synthesis.	HIGH -> Discusses attrition patterns and follow-up durations across included RCTs as part of evidence quality.	HIGH -> Provides a clear conceptual rationale and cites a broad policy and regulatory literature base to support its argument.	Conceptually rigorous critique of systemic framing in gambling policy; not empirical and lacks referral or outcome data.
Abbott et al. 2018	N/A - Referral Type: Helpline -> Self-help support.	HIGH -> Reports quantitative outcomes on gambling severity, financial loss, and psychological distress using validated measures.	HIGH -> Includes follow-up assessments and attrition rates, demonstrating sustained effects over time.	HIGH -> Reports clear inclusion criteria, search strategy, and synthesis methods consistent with scoping review standards.	Transparent scoping review of Gamblers Anonymous as a recovery pathway; highlights engagement barriers but lacks longitudinal data.
Columb & O'Gara 2021	HIGH -> Referral Type: Healthcare internal (GP -> Mental health service).	HIGH -> Reports quantitative outcomes (e.g., accepted, redirected, or rejected referrals and engagement rates).	LOW -> No longitudinal tracking after initial referral outcomes.	HIGH -> Reports participant demographics, recruitment, and analytic methods; clearly describes clinical service settings and sample composition.	Well-reported and methodologically clear cross-sectional survey identifying systemic readiness and referral barriers within community mental health services; contributes valuable evidence on clinician-level pathways and system integration gaps.
Manning et al. 2020	HIGH -> Referral Type: Clinician-initiated referral within community mental health services.	HIGH -> Quantitative results on screening frequency, referral behavior, and perceived barriers are clearly presented and statistically analyzed.	LOW -> Cross-sectional design; no follow-up on referral outcomes or longitudinal effects.	HIGH -> Describes setting, participant population, intervention structure, and qualitative data-collection procedures in detail.	Context-rich qualitative study showing how shelter-based models engage marginalized groups; lacks formal referral definitions and quantitative outcome data.
Nehlin et al. 2016	HIGH -> Referral Type: Primary care screening to brief intervention.	HIGH -> Provides measurable feasibility outcomes (engagement, acceptability, participation rates) and qualitative feedback from caregivers.	LOW -> Pilot phase design; limited follow-up beyond immediate implementation outcomes.	HIGH -> Reports program design, participant demographics, analytic procedures, and data-collection timepoints in detail.	Robust longitudinal study with validated outcomes; highlights partial effectiveness of integrated care but lacks referral or pathway analysis.
Oakes et al. 2020	HIGH -> Referral Type: Helpline -> Treatment.	HIGH -> Reports measurable outcomes related to treatment readiness and emotional stabilization, supported by quantitative and qualitative data.	LOW -> Immediate post-contact outcomes only; no longitudinal follow-up beyond initial helpline intervention.	HIGH -> RCT methods, sample characteristics, randomization procedure, and intervention design are fully reported.	Strong RCT with clear reporting and validated outcomes; focuses on self-initiated digital help-seeking without an external referral component.
Dowling et al. 2019	LOW -> Referral Type: Screening -> Identification only.	HIGH -> Quantitative outcomes (sensitivity, specificity, predictive values) were reported for each screening tool.	LOW -> Primary studies rarely reported follow-up or longitudinal validation; the review explicitly notes this limitation.	HIGH -> Strong methodological reporting of randomization, intervention conditions, and analytic approach.	Methodologically rigorous RCT demonstrating that single-session treatments can achieve comparable outcomes to longer interventions for certain gambling clients; strong design and reporting, but no referral process was examined.

Håkansson et al. 2019	LOW -> Referral Type: Public perception of treatment pathways (no defined referral process).	HIGH -> Quantitative outcomes reported on treatment attitudes, help-seeking intentions, and perceived institutional responsibility.	LOW -> Single-wave cross-sectional survey; no follow-up or longitudinal assessment.	HIGH -> Detailed questionnaire reporting, sample characteristics, and statistical analysis are provided.	Sound cross-sectional study revealing professional knowledge gaps and limited system readiness for adolescent gambling harm; lacks defined referral mechanisms.
Hing et al. 2015	LOW -> Referral Type: Individual help-seeking -> Informal or online support.	HIGH -> Provides quantitative outcomes on help-seeking prevalence, modality differences, and behavioral correlates.	LOW -> Cross-sectional survey; no follow-up or longitudinal assessment.	HIGH -> Clearly describes participant recruitment, data collection, and analytic methods (thematic analysis).	Contextually detailed qualitative study mapping systemic and attitudinal barriers to gambling screening; offers policy insights but lacks a defined referral mechanism.
Ledgerwood & Arfken 2017	LOW -> Referral Type: Within-treatment system assessment (outpatient -> higher-intensity services).	HIGH -> Quantitative findings on perceived need for higher care levels and correlates (e.g., severity, comorbidity) are clearly reported.	LOW -> Cross-sectional design; no longitudinal follow-up or attrition reporting.	HIGH -> Describes inclusion criteria, databases searched, synthesis approach, and methodological quality of included trials with clarity.	Transparent synthesis of RCT-based treatment evidence with consistent outcome reporting; does not examine referral pathways or system linkage.
Månsson et al. 2022	LOW -> Referral Type: Within-system treatment provision (no explicit referral mechanism).	HIGH -> Quantitative outcomes on competence, technique use, and organizational context are reported and statistically analyzed.	LOW -> Cross-sectional design; no longitudinal tracking or follow-up component.	HIGH -> Clear description of sampling, recruitment, and survey instrument; robust national coverage.	National mapping of treatment provision and counselor competence offers organizational insights but lacks referral process analysis or outcome tracking.
Rodda et al. 2015	LOW -> Referral Type: Self-initiated online counseling.	HIGH -> Provides robust quantitative outcomes describing motivational and behavioral subtype distributions with implications for engagement.	LOW -> Cross-sectional analysis with no longitudinal tracking or follow-up component.	HIGH -> Clearly details databases, inclusion criteria, analytic procedures, and meta-analytic methods; transparent and replicable.	High methodological transparency and robust quantitative synthesis of screening accuracy; it does not define or evaluate referral pathways, focusing instead on diagnostic validity.
Sansanwal et al. 2016	LOW -> Referral Type: Professional awareness and training context.	HIGH -> Reports quantitative findings on knowledge, attitudes, and confidence levels; interprets structural implications for system preparedness.	LOW -> One-time survey with no follow-up or longitudinal component.	HIGH -> Clear description of audit methodology, platform selection, and replication of previous protocol.	Transparent audit of operator RG tools with strong metrics and implementation insights; lacks formal referral or healthcare linkage analysis.
Shin et al. 2014	LOW -> Referral Type: Clinic-based treatment cohort.	HIGH -> Quantitative outcomes on treatment delays, medication response, and demographic predictors are clearly presented.	LOW -> Cross-sectional design with limited follow-up reporting on treatment continuation or relapse.	HIGH -> Clear description of survey design, sampling (GPs across six cantons), and response rates.	Transparent cross-sectional design with clear quantitative measures; lacks referral definition and follow-up.
Blank et al. 2021	HIGH -> Referral Type: Healthcare / Support Services Screening -> Brief Intervention or Referral (SBIRT).	HIGH -> Reports measurable evidence of screening uptake, intervention completion, and early referral outcomes across included studies.	N/A -> Limited reporting of follow-up data in the primary studies; attrition not systematically assessed.	HIGH -> Reports recruitment, clinical context, BI procedure, and participant feedback; clear mixed-methods reporting.	Transparent and well-designed pilot study demonstrating the feasibility of delivering brief interventions for gambling in primary care and identifying contextual conditions for successful referral.

Schuler et al. (2016)	HIGH -> Referral Type: Self-help and mutual aid recovery context.	HIGH -> Synthesizes evidence on abstinence, relapse prevention, and psychosocial outcomes, with attention to variability in reported measures.	N/A -> Review-level synthesis; follow-up data not applicable to study design.	HIGH -> multi-source dataset is described clearly with sampling procedures, inclusion criteria, and analytical methods.	Transparent data and robust quantitative measures examining predictors of treatment utilization; clearly identifies personal, social, and structural correlates of help-seeking, but lacks longitudinal tracking.
Achab et al. (2014)	LOW ->The study discusses referral awareness and perceived gaps, but does not define or evaluate an explicit referral process.	HIGH -> Reports measurable indicators (screening frequency, referral awareness, confidence levels).	N/A -> Cross-sectional design; no follow-up or attrition data reported.	HIGH -> Clear description of the co-design process, participant groups, implementation methods, and analytic framework.	Well-executed feasibility pilot offering insight into real-world referral implementation in community and GP settings; includes robust qualitative reporting and linkage evaluation.
Bischof et al. 2014	LOW -> The study examines predictors of treatment utilization but does not define or analyze the structural referral or linkage process that leads to care.	HIGH -> Reports measurable outcomes—treatment utilization rates, gambling severity, and comorbidity indicators—with clear quantitative analyses.	N/A -> Cross-sectional design; no longitudinal tracking or follow-up of participants.	HIGH -> Methods, participant characteristics, and helpline context are described in detail, with mixed-methods procedures clearly outlined.	Clear, well-defined referral model with robust reporting and outcome measures; limited by the absence of follow-up data.
Catania & Griffiths 2021	LOW -> Evaluates operator-based protection features and customer responses but does not define or measure a formal referral process.	HIGH -> Reports measurable indicators (tool availability, staff responses, compliance rates).	N/A -> Cross-sectional audit; no follow-up component.	HIGH -> Describes qualitative design, recruitment, institutional contexts, and thematic analysis with strong methodological clarity.	Contextually detailed systems-level study on integration of gambling services into mental health frameworks; identifies coordination gaps and referral tool limitations.
Rodda et al. 2018	LOW -> Referral Type: Mental health services screening context.	HIGH -> Provides rich qualitative outcomes identifying structural, professional, and perceptual barriers and enablers.	N/A -> Single-point qualitative study with no follow-up phase.	HIGH -> Clearly reports study design, participant roles, sampling approach, recruitment procedures, and analytic method.	Qualitative study offering insight into practitioner attitudes and engagement factors; lacks defined referral mechanisms or quantitative outcome data.
Sanchez et al. 2019	LOW -> Referral Type: Pre-implementation clinical setting.	HIGH -> Reports well-structured qualitative findings on determinants of IBI acceptability and implementation barriers.	N/A -> Single-stage focus group design; no follow-up phase.	HIGH -> Clear qualitative design with description of participants (therapists, professionals), data collection methods, and analytical framework.	Transparent qualitative design identifying key institutional and stigma-related barriers to gambling treatment access; provides strong system-level insights but lacks defined referral mechanisms or outcome measures.
Mfoafo-M'Carthy et al. 2022	HIGH -> Referral Type: Within-system referral (Addiction/Mental Health - PG services).	LOW -> Reports thematic outcomes related to system integration and service coordination rather than quantifiable client-level results.	N/A -> One-phase qualitative study; no longitudinal follow-up component.	HIGH -> Clearly reports participant characteristics, recruitment, instruments, and analytical approach.	Well-described cross-sectional survey identifying structural readiness gaps and unmet service needs in outpatient treatment; informative for system-level planning but without defined referral mechanisms or follow-up data.

Dabrowska et al. 2017	LOW -> Referral Type: Public treatment system -> Addiction/Mental Health Services.	LOW -> Thematic findings describe perceived barriers and redirection patterns but lack measurable outcome indicators.	N/A -> Qualitative cross-sectional interviews; no longitudinal design or follow-up element.	HIGH -> Clearly describes dataset size, sources (national helpline logs), variables, and analytic approach (multivariate regression).	Robust longitudinal dataset with clear operationalization of referral processes and quantitative outcome reporting; represents one of the highest-quality examples of helpline-to-treatment linkage.
Dorey et al. 2022	LOW -> Referral Type: Within-service treatment engagement only.	LOW -> Findings describe practitioner perceptions and themes rather than measurable outcomes or quantified engagement effects.	N/A -> Cross-sectional qualitative interviews; no longitudinal follow-up component.	HIGH -> Systematic review methods (databases, search strategy, inclusion criteria, synthesis process) are clearly described.	Comprehensive and transparent systematic review of healthcare-based screening and referral models; limited longitudinal data from primary evidence.
Fogarty et al. 2018	LOW -> Referral Type: Community engagement -> Informal support or education.	LOW -> Reports descriptive case outcomes and narrative impacts but lacks standardized or quantifiable measures.	N/A -> Case-based design with no longitudinal tracking or evaluation component.	HIGH -> Reports sample characteristics, clinical assessment methods, and analytic procedures with clarity.	Well-reported clinical study offering valuable insights into how age of onset shapes treatment experiences and outcomes in specialized care settings; focuses on predictors rather than referral processes.
Hancock & Smith 2017	LOW -> Referral Type: Conceptual critique (no referral process).	LOW -> Analytical and interpretive; does not include measurable outcomes or empirical testing.	N/A -> Theoretical policy analysis with no participant data or longitudinal design.	HIGH -> Provides clear descriptions of participant composition, recruitment, and analytic approach	Well-reported qualitative study on internet-based intervention acceptability and readiness; lacks referral pathway analysis.
Lopez-Gonzalez et al. 2022	LOW -> Referral Type: Therapy adaptation (no defined referral process).	LOW -> Reports thematic insights into provider experiences rather than measurable outcomes.	N/A -> Single-session focus groups; no longitudinal follow-up or attrition applicable.	HIGH -> Detailed reporting of dataset, variables, modeling techniques, and validation procedures; strong methodological transparency.	Methodologically robust modeling study using real-world data to identify attrition risks; informs service design but does not assess referral processes.
Matheson et al. 2022	LOW -> Referral Type: Shelter-based intake -> Integrated support services.	LOW -> Reports qualitative outcomes (improved stability, reduced gambling, enhanced self-agency) but lacks standardized or quantifiable indicators.	N/A -> Single-phase qualitative evaluation; no longitudinal component or follow-up data.	HIGH -> Fully reports recruitment context, randomization, intervention arms, and analytic methods; transparent in its online design.	Transparent online RCT demonstrating modest efficacy and high attrition among non-help-seeking online gamblers; provides insight into engagement barriers but lacks defined referral or pathway elements.
McDowell et al. 2020	LOW -> Referral Type: Professional competency development (no referral process).	LOW -> Reports consensus-derived competencies qualitatively; lacks quantifiable or clinical outcome measures.	N/A -> Consensus-based design; no participant attrition or longitudinal follow-up applicable.	HIGH -> Describes participant backgrounds, institutional context (public and NGO-based services), focus-group design, and thematic analysis process.	Context-rich qualitative study revealing provider–system mismatches in addressing online gambling harm; offers insight into adaptations but lacks referral or outcome data.
Regan et al. 2022	LOW -> Referral Type: Policy framework (no direct referral process).	LOW -> Reports consensus ratings on implementation feasibility and perceived policy effectiveness; does not include measurable service outcomes.	N/A -> Delphi design with expert participation across structured rounds; no follow-up component relevant.	Survey design, sampling frame, and analytic methods are clearly reported, including respondent demographics and national representativeness.	Transparent and methodologically sound national survey capturing public attitudes toward treatment settings and responsibility framing; provides perception data rather than empirical referral evidence.

Abbott 2017	N/A - Conceptual commentary; no discussion of specific referral mechanisms or pathways.	N/A -> Commentary paper; no measurable outcomes or indicators.	N/A -> Conceptual work.	HIGH -> Clearly reports sample size, analytic methods, and statistical modeling procedures.	Strong quantitative study with clear evidence on helpline-to-treatment referral and follow-through predictors; one of few to empirically examine referral success factors.
Toneatto 2016	LOW -> Referral Type: Self-initiated treatment enrollment.	HIGH -> Robust quantitative outcomes, including gambling frequency, expenditure, DSM-IV symptom reduction, and treatment satisfaction.	HIGH -> Follow-up assessments were conducted across intervention arms to measure sustained change.	N/A -> non-empirical, no data source or sample reported.	Non-empirical conceptual commentary: excluded from quality grading but provides important theoretical context.

Table 6: Rapid quality appraisal of the included manuscripts

Description: The table above summarizes the results of the rapid quality appraisal conducted across all 39 included studies. Each study was labeled as High, Low, or N/A for four core criteria—clarity of referral definition, adequacy of outcome measurement, follow-up reporting, and data transparency—to describe the evidential strength and methodological clarity of the literature base.

Label Definitions:

High: Criterion clearly met and explicitly reported in the study.
Low: Criterion weakly addressed, vague, or not supported by evidence.
N/A: Not applicable due to conceptual or theoretical design.

Appraisal Criteria:

- Clarity of referral or pathway definition
  - High: Referral type clearly defined (e.g., Healthcare/Support Services Screening -> Brief Intervention or Referral [SBIRT]).
  - Low: Referral is mentioned vaguely or without a clear definition.
  - N/A: Paper is purely conceptual or theoretical in nature.

- Adequacy of outcome measurement
  - High: Reports measurable outcomes (e.g., referral completion, engagement rates, treatment uptake).
  - Low: Only perceptions, barriers, or intentions are reported.
  - N/A: Study is not designed to measure outcomes (e.g., theoretical or policy-focused papers).

- Reporting of attrition or follow-up
    - High: Provides information on drop-out, completion rates, or follow-up response.
    - Low: No attrition or follow-up information is provided.
    - N/A: Cross-sectional or non-participant-based design where follow-up is not applicable.

- Transparency of the data source and sampling
    - High: Clearly describes data source and sampling strategy (e.g., “clinic records,” “national helpline users,” “survey of therapists”).
    - Low: The empirical basis is unclear or weakly described.
    - N/A: Not an empirical study (e.g., commentary, editorial, or conceptual framework).
